# Contemporary Genetic Structure, Phylogeography and Past Demographic Processes of Wild Boar *Sus scrofa* Population in Central and Eastern Europe

**DOI:** 10.1371/journal.pone.0091401

**Published:** 2014-03-12

**Authors:** Szilvia Kusza, Tomasz Podgórski, Massimo Scandura, Tomasz Borowik, András Jávor, Vadim E. Sidorovich, Aleksei N. Bunevich, Mikhail Kolesnikov, Bogumiła Jędrzejewska

**Affiliations:** 1 Institute of Animal Science, Biotechnology and Nature Conservation, University of Debrecen, Debrecen, Hungary; 2 Mammal Research Institute, Polish Academy of Sciences, Białowieża, Poland; 3 Department of Science for Nature and Environmental Resources, Sassari, Italy; 4 Institute of Zoology, National Academy of Sciences of Belarus, Minsk, Belarus; 5 State National Park Belovezhskaya Pushcha, Brest Oblast, Kamenec Raion, Kamenyuki, Belarus; 6 Luhansk Taras Shevchenko National University, Luhansk, Ukraine; Natural History Museum of Denmark, Denmark

## Abstract

The wild boar (*Sus scrofa)* is one of the most widely distributed mammals in Europe. Its demography was affected by various events in the past and today populations are increasing throughout Europe. We examined genetic diversity, structure and population dynamics of wild boar in Central and Eastern Europe. MtDNA control region (664 bp) was sequenced in 254 wild boar from six countries (Poland, Hungary, Belarus, Ukraine, Moldova and the European part of Russia). We detected 16 haplotypes, all known from previous studies in Europe; 14 of them belonged to European 1 (E1) clade, including 13 haplotypes from E1-C and one from E1-A lineages. Two haplotypes belonged respectively to the East Asian and the Near Eastern clade. Both haplotypes were found in Russia and most probably originated from the documented translocations of wild boar. The studied populations showed moderate haplotype (0.714±0.023) and low nucleotide diversity (0.003±0.002). SAMOVA grouped the genetic structuring of Central and Eastern European wild boar into three subpopulations, comprising of: (1) north-eastern Belarus and the European part of Russia, (2) Poland, Ukraine, Moldova and most of Belarus, and (3) Hungary. The multimodal mismatch distribution, Fu's Fs index, Bayesian skyline plot and the high occurrence of shared haplotypes among populations did not suggest strong demographic fluctuations in wild boar numbers in the Holocene and pre-Holocene times. This study showed relatively weak genetic diversity and structure in Central and Eastern European wild boar populations and underlined gaps in our knowledge on the role of southern refugia and demographic processes shaping genetic diversity of wild boar in this part of Europe.

## Introduction

The wild boar *Sus scrofa* originated in South-East Asia, where the genus *Sus* differentiated about 3 million years ago and from where *S. scrofa* spread throughout Asia, Europe and North Africa [1]. Wild boars appeared in Europe 1.5 to 0.4 million years ago, depending on whether estimates are based on archaeological or molecular data [2]. The present distribution of wild boar in Europe was primarily shaped by the late Pleistocene glaciations that forced wild boars to take refuge in southern areas (the Iberian Peninsula and south-western France, the Italian Peninsula, and the Balkan region from Greece to Croatia and Slovenia [3]) from where the species re-colonised the continent [2], reaching as far north as 60°N in western Russia [4].

It is, however, not clear which of these sources of refuge contributed the most to the re-establishment of the current population in Europe, especially in its eastern part. Continental Europe is populated by wild boar belonging to two major haplogroups; clade E1 is widespread throughout the entire continent and clade E2 is restricted to the Italian Peninsula, Sardinia and Croatia [5], [2]. The clade E1 is not only the most widespread but also the most diverse, with two widely distributed clusters: A-side, which is common and possibly dominating in the region from Italy and France to Germany and Austria, but is rare in the Balkans and Iberian Peninsula, and C-side, which is widespread in Europe, and proliferates in two regions – Iberia and Central Europe (Poland, Hungary) – reaching nearly 90% frequency among wild boar [2]. Other clusters belonging to E1 haplogroups (W1-W6) have recently been discovered in the Southern Balkan region (Greece and SE Bulgaria) and their occurrence seems to be restricted to that area [6].

However, the genetic affinity of wild boar populations from east and northwest part of Europe has not yet been thoroughly studied. [7]. Thus phylogenetic affiliation of wild boar from the European part of Russia, Ukraine, Belarus, and most of Poland remains unknown. This area could harbour animals belonging to a largely homogenous cluster C or it could also have a substantial admixture of haplotypes from clusters W1 and W2 originating from the Balkan region [6].

In addition to the impact of past glaciation, the contemporary phylogeographic profile of wild boar in Europe could have been affected by more recent events. In Eastern Europe, demographic decline in wild boar occurred in the 17^th^–19^th^ centuries when the combined effects of climate cooling (Little Ice Age) and overexploitation by humans reduced the population numbers and distribution in many regions [4], [8]. The species became temporarily extinct in some countries, e.g. the Baltic States and the Czech Republic [8]. Other populations (e.g. in Poland, Hungary) were reduced [9]–[10] or restricted to southern peripheries (Western Russia [4]). From the end of 19^th^ century, re-colonization from populations in Poland, Hungary, Slovakia and possibly the Ukraine started. After World War II, the density and geographical distribution of wild boars increased throughout most of Europe and by the middle of the 20^th^ century wild boar populations were restored [11]–[12], [4].

In the last 50 years, a rapid increase in wild boar numbers was observed across Europe [11]. In Eastern European countries populations have increased five to tenfold [8]. Currently, wild boars are widely distributed in Europe with population densities following the latitudinal gradient and declining by two orders of magnitude northwards [13].

The most important event in shaping the pattern of genetic diversity of Western and Central European wild boar was the last glaciation, which was followed by a sudden demographic and spatial expansion of the populations [2], [5]. On the contrary, human-induced gene flow (translocations, hybridisation) and demographic declines appeared to have had higher influence on the genetic make-up of the current populations than it was previously considered. Twenty-seven percent of the wild boar studied in Luxembourg had introgession of domestic pig mtDNA, while Ireland turned out to have been colonized by captive pigs [14]–[15].

The objective of our study was to characterize genetic diversity, structure, and phylogenetic relationships among Central and Eastern European populations of wild boar using the mitochondrial (mtDNA) control region. Specifically, we aimed at: (1) describing mtDNA variability with a reference to haplotype diversity observed in the rest of Europe and Asia, (2) determining the genetic structure of the populations and (3) understanding if currently observed genetic diversity and structure have a signature of past, post-glacial demographic expansion. We used wide-range geographic sampling over six countries of Central and Eastern Europe to give the first comprehensive genetic characteristics of the wild boar populations from this part of Europe.

## Materials and Methods

### Sampling and laboratory analysis

**Figure 1:**
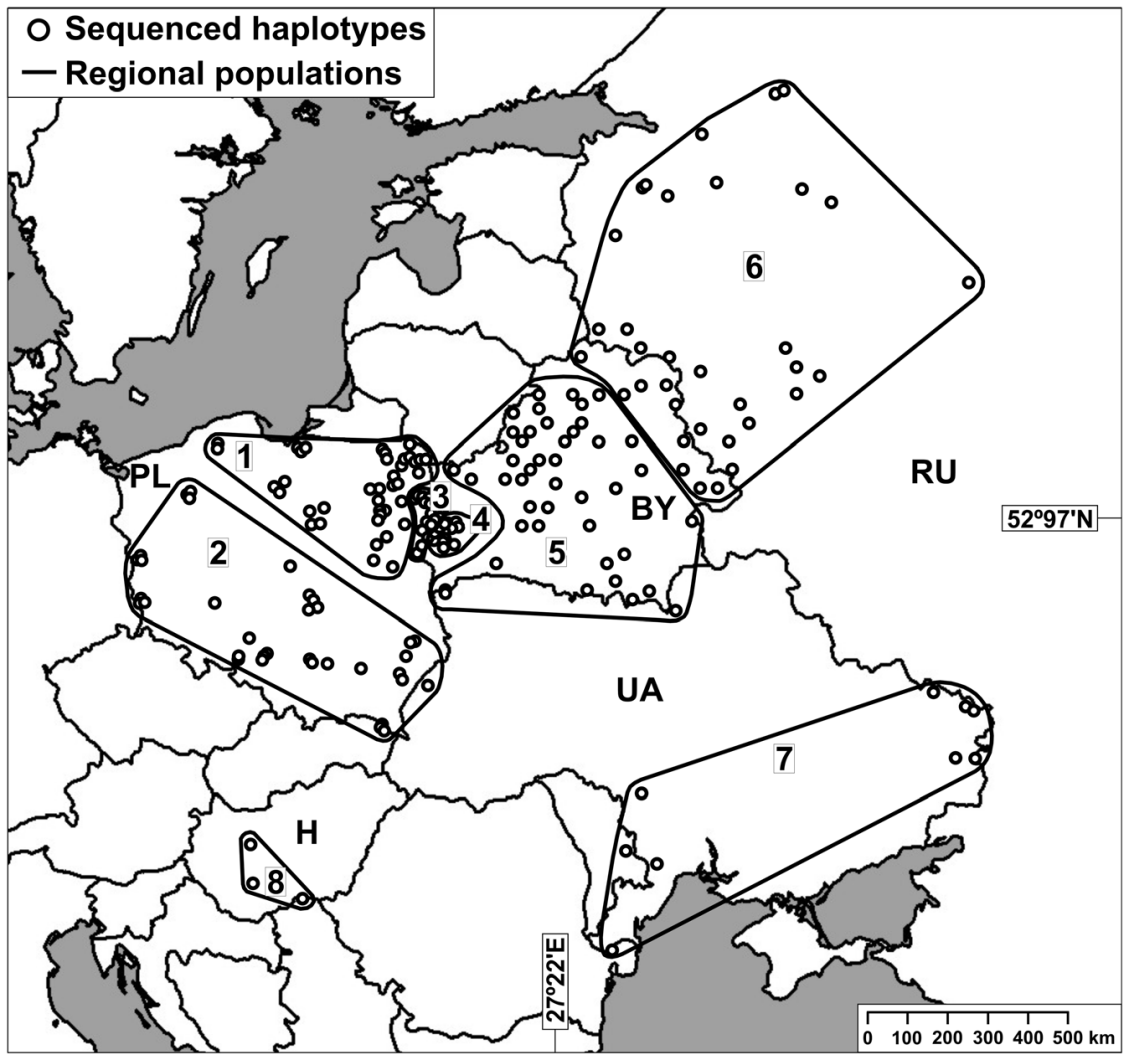
Maps showing distribution of the wild boar sampling sites. Division of the samples into 8 groups is based on geographic location.

In total, 254 tissue samples were collected in 2007–2010 in six Central and East European countries (Belarus 74, Hungary 15, Poland 118, Ukraine 15, Moldova 1, European part of Russia 31; Figure 1). Fresh muscle or skin fragments were sampled from legally hunted unprotected wild boars and either stored in plastic tubes (5–30 ml) filled with 96% alcohol or kept frozen at the temperature of −20°C. Animals were not shot only for the purpose of this study. The study did not involve collection of samples from live animals. Ethics statement was not required. Samples from the different countries were obtained from collaborators, hunters and used with their permission. They collected samples in accordance with their national regulations on wild boar management. All wild boars were legally hunted by licensed hunters.

Genomic DNA was extracted using the Qiagen DNeasy Blood and Tissue Kit following the manufacturer's protocol. A 664 bp fragment of the mitochondrial control region was amplified by the polymerase chain reaction (PCR) using two primers: forward Ss.L-Dloop: CGCCATCAGCACCCAAAGCT [16] and reverse PrR: ACCATTGACTGAATAGCACCT [17]. PCRs were carried out in a total volume of 10 μl, containing 9 μl Hot Star Taq Master mix (Qiagen), ca. 100 ng DNA and 0.5 μM of both forward and reverse primers under the following conditions: 95°C for 15 min and 35 cycles of 94°C for 1 min, 55°C for 1 min and 72°C for 1 min and finally an extension step at 72°C for 10 min. The amplified products were purified by Clean-up kit (A&A Biotechnology, Gdynia, Poland). Sequencing reactions were carried out using the forward primer Ss.L-Dloop and the ABI Prism BigDye Terminator v3.1 Cycle Sequencing kit. The sequencing was performed using an ABI 3100 automated DNA analyzer. Sequences were aligned using the BioEdit 7.0 software [18]. Analyses were performed at the laboratory of the Mammal Research Institute Polish Academy of Sciences, Białowieża, Poland.

### Statistical analysis

Haplotype and nucleotide diversities, and the number of polymorphic sites were calculated with DnaSP 5.00 [19].

The best-fit model of nucleotide substitution was selected across 288 candidate models using jModeltest 2.1.4 [20] on the alignment of wild boar sequences, plus two sequences of *Sus barbatus* as outgroups (Genbank accession numbers AJ314540 and GQ338953). The best model resulted the HKY model [21] with gamma-distributed (G) rate variation across sites, based on the corrected Akaike Information Criterion (-lnL  =  1531.56, AICc weight  =  0.777). To place our results in a broader phylogeographical context, haplotypes detected in our study were compared to those obtained from 598 wild boar and domestic pig sequences available in GenBank ([5]–[6], [16], [22]–[34]; Table S1). All haplotypes were then combined into a Bayesian phylogenetic tree, built in MRBAYES v. 3.2 [35], and into a MJ network, which was constructed using NETWORK version 4.6.0.0 [36]. For these analyses all sequences obtained in this study were shortened from the original size of 664 bp to 411 bp to allow for comparison with the sequences available at GenBank. The following settings were used for the Bayesian phylogenetic tree: HKY+G model of sequence evolution, two runs each composed by one cold and three heated Monte Carlo Markov Chains, 1,000,000 generations of chain length, sampling every 1000 generations. The first 25% of the sampling trees and estimated parameters were discarded as burn-in. Convergence was monitored by the decrease in standard deviation of split frequencies and the Potential Scale Reduction Factor (PSRF) associated to the model parameters. The final consensus tree was drawn in FigTree 1.4.0 [37].

For spatial analyses of the population structure, we divided our sampling area into 8 regions: (1) northern Poland, (2) southern Poland, (3) the Polish part of the Białowieża Forest, (4) the Belarusian part of the Białowieża Forest, (5) most of Belarus; (6) eastern Belarus and western Russia, (7) Ukraine and Moldova, and (8) Hungary (Figure 1). Samples from the Białowieża Forest (Polish and Belarusian parts) were separated in two because of the border fence erected in 1981 that could have acted as barrier to gene flow.

We assessed population structure of mtDNA using spatial analysis of molecular variance – SAMOVA [38], which calculates the genetic structure based on the genetic data and geographic location of populations. SAMOVA requires a priori definition of the number of groups (K). Thus, the analysis was performed for K ranging from 2 to 8. We computed the genetic distances among subpopulations found by SAMOVA using Arlequin 3.1 [39].

We used Arlequin 3.1 to test the hypothesis of a past population expansion by calculating Fu's and Tajima's statistics [40], [41] and testing their significance over 1000 permutations. In addition, deviations from a model of population expansion were evaluated by computing statistical significance of sums of squared deviation (SSD) and Harpending's raggedness index (r) over 1000 simulated samples of pairwise nucleotide differences. To estimate variation in female effective population size over time from mtDNA sequences, a Bayesian skyline plot (BSP) model with standard Markov chain Monte Carlo sampling procedure (MCMC), strict molecular clock and 1.36×10^−8^ mutation rate, 1,5 years generation time was used in BEAST 1.6.1. [42], [43]. The analysis was performed using all 254 sequences from this study. The Bayesian skyline plot represents population size changes over time, inferred with mtDNA and the assumed mutation rate. The X-axes are time in millions of years. Y-axes are mean effective population size in millions of individuals divided by generation time on a log scale. Areas between two dotted lines encompass 95% highest posterior density (HPD). The MCMC analysis was run for 10 million generations. The first million was discarded as burn-in (samples were drawn only from the stationary distribution) and parameter values were sampled every 1000 generations. It was necessary in order to assess convergence and confirm that the effective sample sizes were adequate (>200), demonstrating that the MCMC had enough long ran to give valid estimates for the parameters. The BEAST-run was visualized with Tracer version 1.5 (MCMC Trace Analysis Tool) [44].

## Results

### mtDNA variation and genetic structure of wild boar populations

Our alignment (664 bp) of 254 wild boars from Central and Eastern Europe yielded 43 polymorphic sites (Table S2). They represent 6.17% of the total number of sites and include one indel. The average nucleotide composition of all sequences was 25.30% C, 26.90% T, 33.65% A and 14.15% G. The mean nucleotide diversity for all samples was 0.003 ± 0.002 (mean ± SD).

We identified 16 haplotypes, and called them H1-H16 (Table 1., Genbank accession numbers: KF258877-KF258892). Six of them corresponded to haplotypes, which were described earlier [29], [30]: haplotypes A corresponds to H3, C corresponds to H1, BA corresponds to H2, E corresponds to H6, EJ corresponds to H14 and BC corresponds to H16. The mean haplotype diversity was 0.714 ± 0.023. The most frequent haplotype (H1, 48% in the whole sample) was dominating or very common in all regions: from 24% in region 6 (NE Belarus and European part of Russia) to 67% in region 7 (Ukraine) (Table 1). The second most common haplotype, H2 (19% among all wild boar) dominated in region 6 (39%).

**Table 1:** Number of detected haplotypes in regional populations of wild boar (*Sus scrofa*) in Central and Eastern Europe (1: northern Poland, 2: southern Poland, 3: Polish part of the Białowieża Forest, 4: Belarusian part of the Białowieża Forest, 5: Belarus; 6: eastern Belarus and western Russia, 7: Ukraine, 8: Hungary).

Haplotype	Regional populations								Total number	%
	1	2	3	4	5	6	7	8		
H1	21	14	22	17	20	10	8	9	121	47.6
H2	5	8	8	7	3	16	2	-	49	19.3
H3	1	1	-	2	6	1	1	-	12	4.7
H4	-	-	-	1	-	-	-	-	1	0.4
H5	10	3	6	1	6	-	-	-	26	10.2
H6	6	8	-	1	1	8	-	3	27	10.6
H7	-	1	-	1	-	1	-	1	4	1.6
H8	-	3	-	-	-	2	1	-	6	2.4
H9	-	-	-	-	-	-	-	1	1	0.4
H10	-	-	-	-	-	-	-	1	1	0.4
H11	-	-	-	-	1	-	-	-	1	0.4
H12	-	-	-	-	1	-	-	-	1	0.4
H13	1	-	-	-	-	-	-	-	1	0.4
H14	-	-	-	-	-	1	-	-	1	0.4
H15	-	-	-	-	-	1	-	-	1	0.4
H16	-	-	-	-	-	1	-	-	1	0.4
Total number	44	38	36	30	38	41	12	15	254	
%	17.3	15.0	14.2	11.8	15.0	16.1	4.7	5.9		100

For detailed location of the populations see Figure 1.

**Figure 2:**
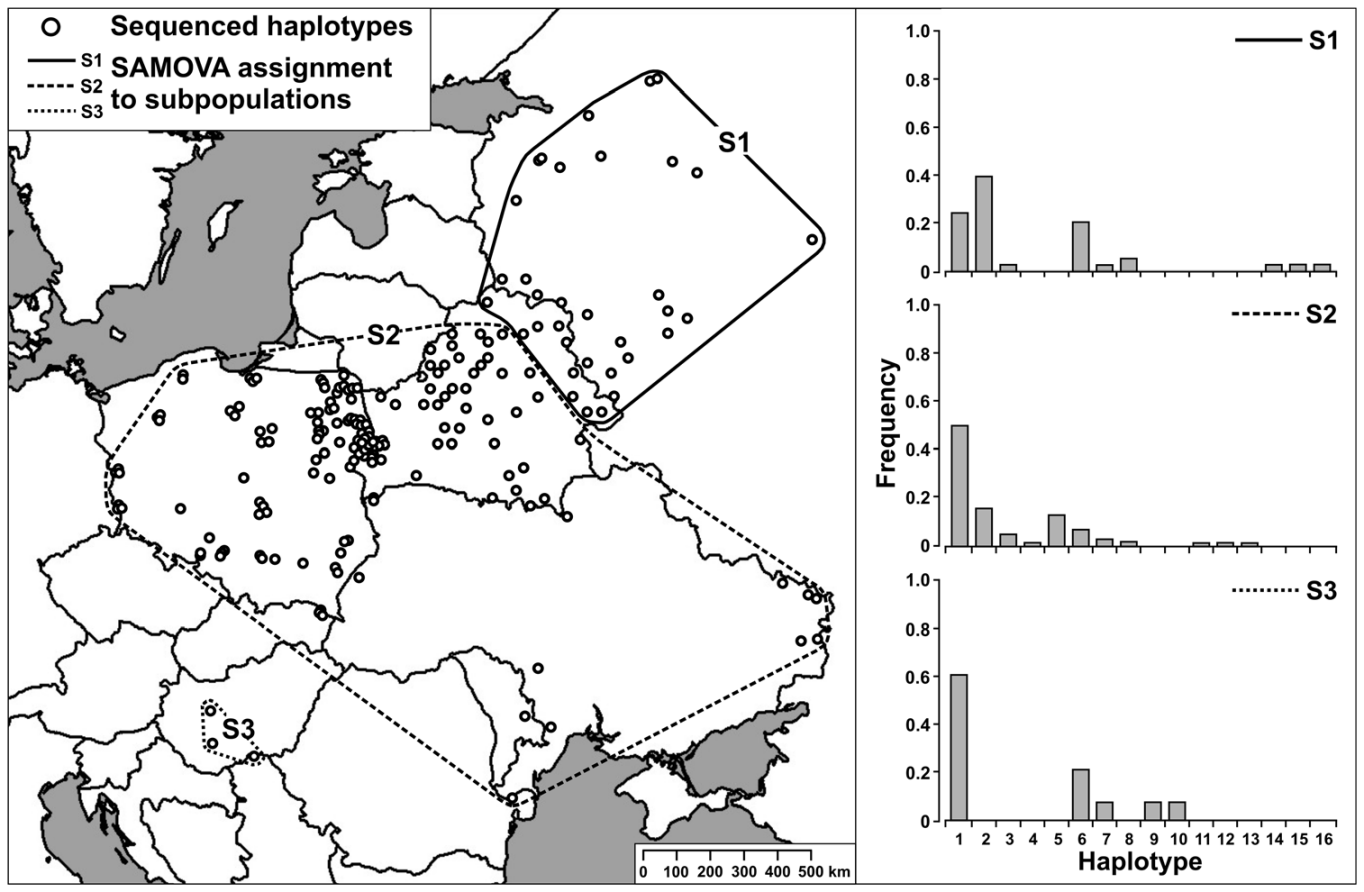
Distribution and haplotype frequencies of three subpopulations determined by SAMOVA.

Optimal spatial structure of the analysed sequences consisted of three subpopulations within the sampling region (Figure 2). Although results of SAMOVA indicated significant genetic differentiation Φ_CT_ for all structuring scenarios, except for K = 2, genetic differentiation among populations within groups Φ_SC_ was the lowest and significant for K = 3 (Table S3). Group S1 (eastern Belarus and western Russia) showed significantly higher genetic distance compared to all other samples (Φ_ST_  =  0.082, *P*<0.001). All other pairwise comparisons were insignificant. This is an effect of the low number of shared haplotypes between S1 and other groups and the dominance of H2 within group S1. Table 2 gives mtDNA variability and diversity parameters for subpopulations determined with SAMOVA.

**Table 2:** Basic parameters of genetic (mtDNA) variability of wild boar subpopulations determined by spatial analysis of molecular variance (SAMOVA) in Central and Eastern Europe.

Parameter	Subpopulation			Total
	S1	S2	S3	
Sample size	41	198	15	254
No. of haplotypes N*_h_*	9	11	5	16
No. of polymorphic sites	34	9	8	41
Haplotype diversity *Hd*(SD)	0.763 (0.042)	0.683 (0.029)	0.629 (0.125)	0.714 (0.023)
Nucleotide diversity *π* (SD)	0.005 (0.003)	0.002 (0.001)	0.002 (0.002)	0.003 (0.002)

### Phylogeographic patterns and past demographic processes

**Figure 3:**
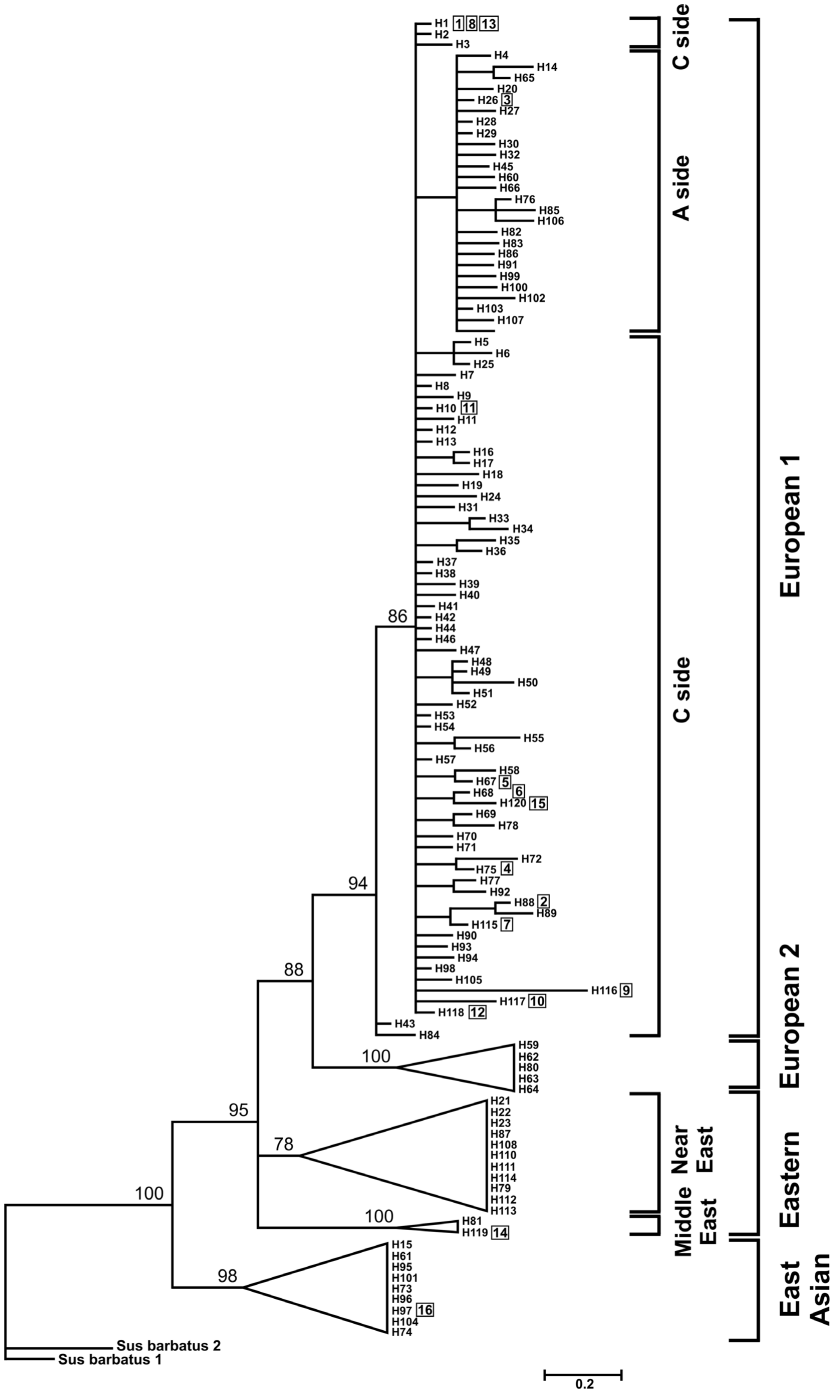
Bayesian phylogenetic tree of 120 haplotypes based on the sequences from the studied Central and Eastern European wild boars (254 sequences) and 598 GenBank sequences from previous studies. Haplotypes detected in this study are marked in squares. Note: due to shortening of our analysed mtDNA fragment from 664 to 411 bp (to allow for comparison with previous studies), 3 haplotypes from this study were collapsed to one single haplotype (see Table S1 for details).

**Figure 4:**
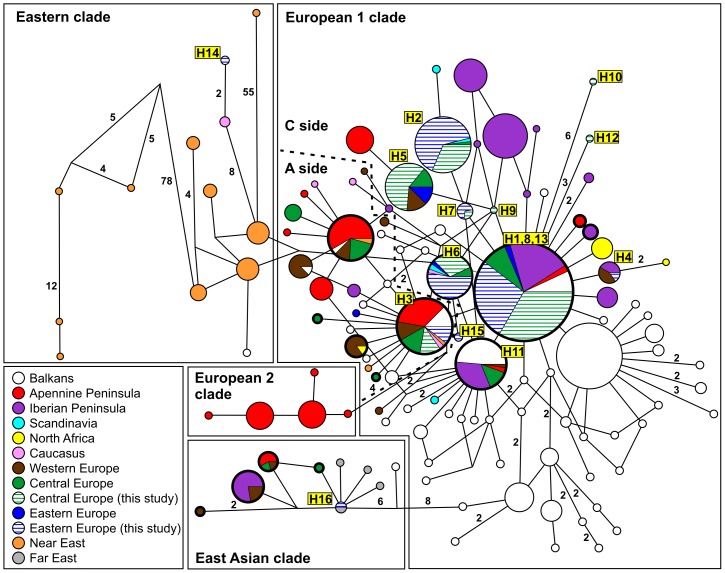
The median-joining network of the haplotypes obtained with 598 wild boar and domestic pig mtDNA sequences from GenBank and 254 wild boar sequences from this study. The size of each circle is proportional to the haplotype frequency. Colours represent regions of sequence origin. European samples are grouped into Eastern Europe (European part of Russia, Belarus, Ukraine, Moldova, Romania, Serbia), Central Europe (Germany, Austria, Switzerland, Slovenia, Hungary, Poland, Czech Republic, Slovakia, Denmark), and Western Europe (Belgium, France, Netherlands, United Kingdom). For more details on countries included in the regions see Table S1. Thick-line circles show presence of domestic pig sequences. Numbers on the lines indicates the number of mutations (no number indicates single mutation).

A Bayesian phylogenetic tree and median-joining network were constructed using our sequences and 598 wild boar and domestic pig sequences from GenBank (Figures 3 and 4). Two of 16 haplotypes were lost due to the reduction in sequence length from 664 to 411 bp. Twelve of 14 haplotypes left in our study grouped with the earlier known European haplogroup E1, and the 2 remaining haplotypes (both recorded in Russia) grouped with the East Asian and Near East haplogroups, respectively. Among E1 sequences, only H3 belonged to the European A cluster (E1-A), and all others belonged to the European C cluster (E1-C).

**Figure 5:**
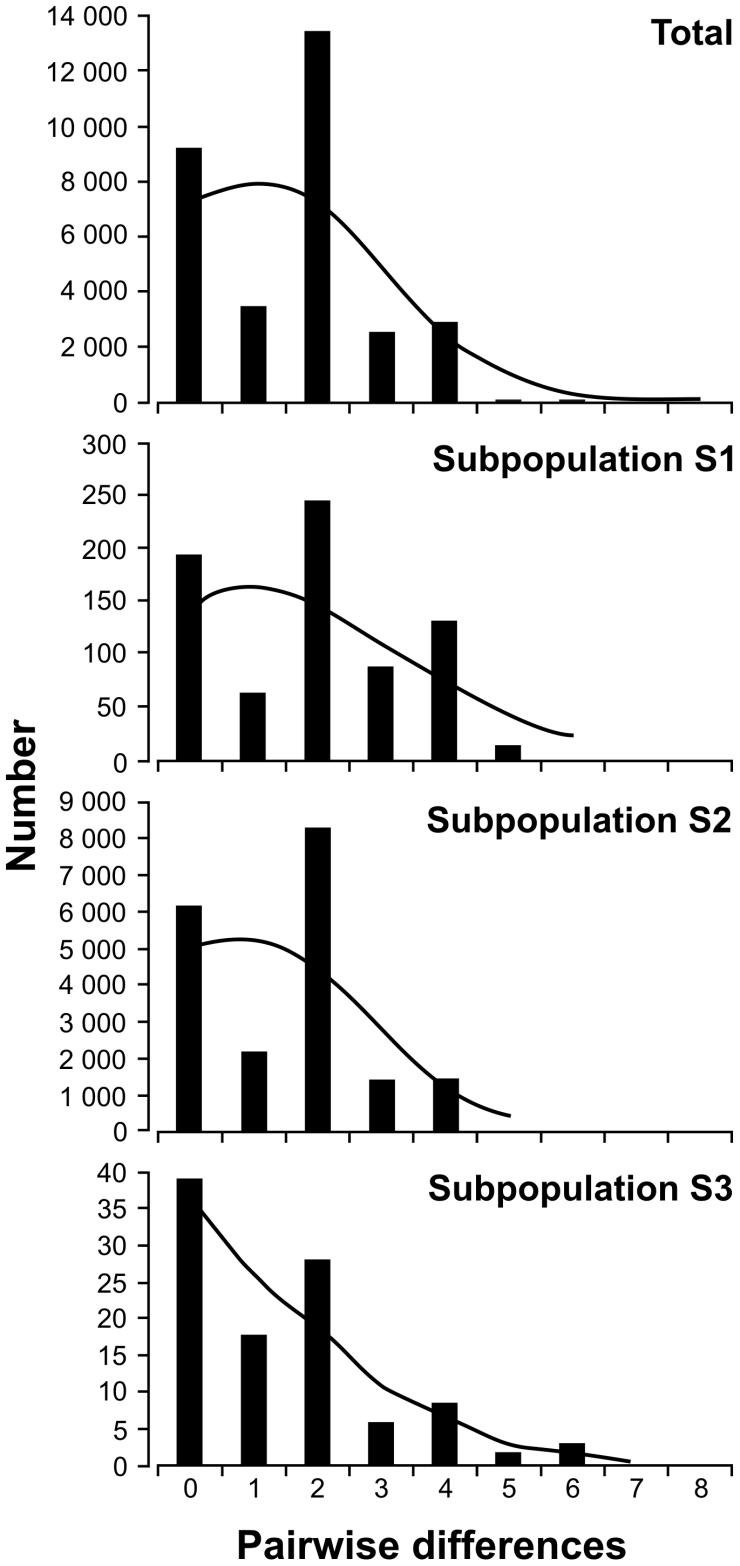
Observed (bars) and simulated (line) mismatch distributions of the mtDNA haplotypes found in this study, in the whole sample (total) and in three subpopulations determined by SAMOVA.

Overall, we obtained negative and non-significant Fu's Fs value and negative non-significant Tajima's D value (Table 3; two alien haplotypes excluded), which suggest no demographic expansion or bottleneck. Harpending's raggedness index was positive and significant at P ≤ 0.05, which shows weak support for past expansion (Table 3). For all samples, as well as for the subpopulations, the mismatch distribution (Figure 5) was ragged and multimodal, which suggests no recent population expansion or bottlenecks.

**Table 3:** Values of neutrality tests (Fs and D), sums of squared deviation (SSD) and Harpending's raggedness index (r) for Central and Eastern European wild boar mtDNA control region sequences.

Parameter	Subpopulation			Overall population	Overall population (haplotype H14 and H16 excluded)
	S1	S2	S3		
Fu's Fs	0.692	−1.900	−0.539	−3.783	−3.348
Tajima's D	−**1.902** *	−0.088	−1.459	−**2.069** **	−0.877
Sum of squareddeviation (SSD)	0.038	0.074	0.019	0.068	0.071
Harpending'sraggedness index	0.137	**0.272** *	0.099	**0.250** *	**0.258** *

*P≤0.05;

**P≤0.001

Significant values are in bold.

**Figure 6:**
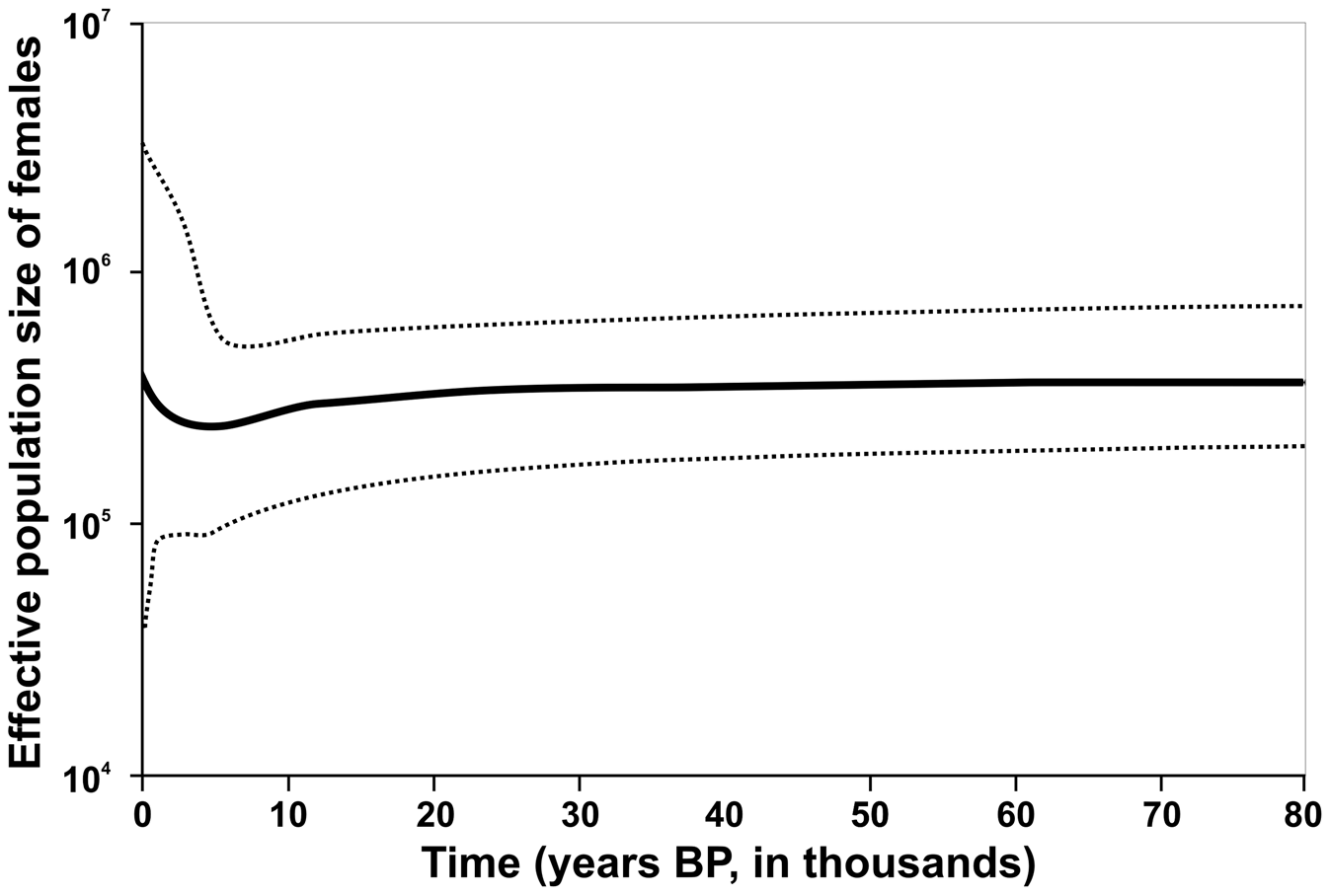
Bayesian skyline plots showing effective population size of wild boar over time in Central and Eastern Europe. Median estimates are shown as solid thick line, 95% highest posterior density (HPD) intervals are represented by dotted lines.

Analysis of the prehistorical population size dynamics in Central and Eastern Europe showed slowly declining population number and a sudden recent increase (Figure 6). The absence of a fall during around 20 000 years BP would mean no evidence of bottleneck during the LGM. The skyline plot indicated that the history of the present population in the studied area started after the LGM, when the wild boars were re-established from southern refugia.

## Discussion

### Mitochondrial genetic variability and structure

We present the first comprehensive data on mtDNA diversity and genetic structure of the wild boar populations in Central and Eastern Europe. In our sample of 254 wild boar from six countries, we detected a total of 16 haplotypes. Other studies revealed 16 haplotypes in 98 Italian wild boar and only 7 haplotypes in 47 other European countries (excluding Italy) [5]. Fifteen haplotypes were detected in 47 Central-European wild boar [32]. Among 129 animals from the Iberian Peninsula [45], 14 haplotypes were detected. Strikingly, 67 wild boar samples from Tunisia yielded only 3 haplotypes [46].

The haplotype diversity for our studied populations (0.714) was lower than those reported for two European wild boar studies [5], [32] (0.902 and 0.910, respectively) but higher than that for the Tunisian wild boar (0.550; [46]). The nucleotide diversity for the overall population (0.003) was lower than in wild boar from Central-Western Europe (0.006; [32]), Europe outside Italy (0.005; [5]), East Asia (0.004; [34]), and the Southern Balkan region (0.011; [6]).

Based on data from 38 sampling locations in Europe (Russia excluded), spatial variation in haplotype diversity of wild boar was analysed [47]. Diversity hot spots were found in south-eastern Spain and southern France, in Greece, and in southern Italy – in accordance with the location of LGM refugia known from fossils records [3]. The least diverse population occurred in north-eastern France, the Netherlands, Germany and Denmark. Interestingly, in Eastern Europe, the haplotype diversity of wild boar was moderate and seemed to increase towards the East, yet no data from Russia were included in the analysis [47]. Our study showed that, indeed, mtDNA diversity of wild boar increased towards east (Hd  =  0.763 in NE Belarus and NW Russia). However, our sampling in the European part of Russia covered only a portion of the wild boar range [7]. Therefore, more extensive and large-scale data are needed from Eastern Europe to elucidate the spatial pattern of wild boar genetic diversity. Our study showed a weak genetic structure of the studied populations with 95% of individuals belonging to lineage C of European haplogroup E1. Only 12 of our studied individuals (5%), belonged to the E1-A lineage from Central-Western Europe and Italy [2] and they all shared the same haplotype (H3). The C-side haplotypes existed throughout Europe before the Last Glacial Maximum (LGM) [6]. During the LGM wild boar populations contracted to various refugia. The European wild boar distribution during the LGM (23000–16000 years BP) was restricted to the Iberian Peninsula, southwestern France, the Italian Peninsula and the Balkans, from Greece northwards to Croatia and Slovenia [2]. A study about wild boar phylogeography in Greece and southern Bulgaria, found several new clusters of haplotypes (within E1 haplogroup) with the occurrence geographically restricted to various regions, which suggested the presence of separate refugia in the southern Balkans [6]. They hypothesized that, after LGM, Central and Eastern Europe was repopulated by wild boar from the Balkan refugium (north of Greece) by the process of ‘leading edge’ dispersal [48]. However, our results neither support nor reject this hypothesis clearly. Wild boar from northern Greece and southern Bulgaria (supposed source population for contemporary wild boar populations in regions north and east of Carpathians) present a diverse mixture of haplotypes belonging to 5 different clusters within the E1 haplogroup, whereas nearly all wild boar in our studied area belonged to one cluster (C). There is, however, a large spatial gap (most of Bulgaria, Romania, and former Yugoslavia), where no data on wild boar mtDNA diversity are available. This is a crucial area to be studied in order to answer questions of postglacial routes of colonization of Eastern and Central Europe by wild boar. Furthermore, a question remains about the possible LGM refugial areas for wild boar in south-eastern regions of Europe, such as contemporary Moldova, Crimea Peninsula (Ukraine) and southern regions of Russian plains. Although only a few wild boar fossil remains have so far been found in these areas, patches of suitable habitats (including broadleaved forest patches) occurred in that region during the LGM [49]. One very common haplotype may represent an ancient lineage that had survived in the presumed eastern refuge and then expanded in Eastern Europe.

In our study, we found one individual belonging to Near East haplogroup and one from East Asian haplogroup (both in Russia). Most probably, these two are signs of past translocations. Twenty-two individuals from northern Caucasus (where Near East haplotypes may occur; [30]) were released in 1971 in forests near Novgorod [50]. About 700 wild boar from various locations in Western Europe and the Russian Far East were released within a 150-200-km radius from Moscow between 1935 and 1967 [51].

### Phylogeographic patterns and past demographic processes

Populations that have gone through a recent expansion show a smooth and unimodal mismatch distribution, short internal branches, weak bootstrap values in a NJ tree, and a star-like structure on a network tree [40]. Fu's Fs test is highly sensitive to demographic expansion, which results in large negative Fs values, whereas the significant Tajima's D value could be a sign of population expansion and bottleneck [41], [52], [53]. The high and non significant raggedness index support the past expansion. The mismatch distribution of our total dataset was not consistent with a recent population expansion and was similar to the Italian population (clades E1 and E2) [5]. The multimodal distribution indicated that no relevant demographic fluctuation have occurred over a long time [54]. The Bayesian skyline plot of wild boar data from Central and Eastern Europe did not suggest a sudden drop in population size in coincidence with LGM followed by a population expansion.

The results of our analyses of mtDNA are seemingly contradictory to data obtained from genome sequencing [55]. They revealed that contemporary wild boar populations from Italy and Holland both suffered a very strong drop in numbers that had began about 60000 yrs BP and reached minimum size during the LGM (∼20000 yrs BP). The bottleneck lasted for the following 10000 yrs after LGM [55]. Also, based on analyses of mtDNA, Italian authors stated that despite the recent demographic changes experienced by European wild boar populations, the postglacial demographic expansion was the main signature on the genetic diversity of all European wild boar, except for the Italian populations [5]. The latter result is again in disagreement with authors [55], who found evidence for a bottleneck in the Italian population. Also, fossil data clearly indicated that during the LGM wild boar geographic range was severely restricted to the Iberian Peninsula, southwestern France, the Italian Peninsula and the Balkans (from Greece northwards to Slovenia and Croatia) [3].

Thus, our results did not exactly fit expectations. Therefore, to fully understand the history of wild boar populations in Europe, we need (1) wider sampling, especially to cover the critical areas of Dinaric – northern Balkan region and southern parts of Ukraine and European Russia, which are candidate regions for the LGM refugia of the contemporary Eastern European wild boars, and (2) applying additional genetic tools such as microsatellite markers and genome sequencing to study wild boar populations at pan-European scale

## Conclusions

1. Central and Eastern wild boar mainly belong to the European E1-C lineage (94% of studied individuals) and only 5% of individuals represented the E1-A lineage. Two animals from Russia had haplotypes typical of East Asian and Near East lineages, and they most likely were traces of past translocations.
2. SAMOVA suggested three genetic subpopulations of wild boar, comprising: (1) north-eastern Belarus and the European part of Russia, (2) Poland, Ukraine, Moldova and most of Belarus, and (3) Hungary.
3. The multimodal mismatch distribution, Fu's Fs index, and Bayesian skyline plot and the occurrence of many shared haplotypes among the populations did not show evidence for strong demographic fluctuations in wild boar numbers in the Holocene and pre-Holocene times.
4. To fully understand the history and to determine the LGM refugia of the extant populations of wild boar Central and Eastern European, it is essential to sample the Dinaric – northern Balkan region, southern Ukraine, and southern portions of Russian Plains, and to study the genetic profile of wild boar by means of microsatellite loci and genome sequencing.

## Supporting Information

Table S1 **List of the wild boar and domestic pig mtDNA sequences used in this study.** Sequences were downloaded from GenBank or obtained by the authors of this study. Clades: A - East Asia, E1 - European 1, E2 - European 2, NE and ME - Eastern (Near East and Middle East, respectively). In European 1 (E1) clade, two sides are distinguished: (A) and (C).
(PDF)

Table S2 **The haplotypes of wild boar** ***Sus scrofa*** **detected in this study and variable nucleotide positions in relation to a reference sequence from GenBank, accession no. AJ002189.**
(DOC)

Table S3 **Results of SAMOVA, Φ_CT_: fixation index among groups; Φ_SC_: fixation index among populations within groups; Φ_ST_: fixation index within populations.**
(DOC)
