# Supplementary material for: Contemporary Genetic Structure, Phylogeography and Past Demographic Processes of Wild Boar Sus scrofa Population in Central and Eastern Europe
Source: PLoS One. 2014 Mar 12;9(3):e91401. doi: 10.1371/journal.pone.0091401 (PMC3951376; doi:10.1371/journal.pone.0091401)
Supplement: Table S1 — List of the wild boar and domestic pig mtDNA sequences used in this study. Sequences were downloaded from GenBank or obtained by the authors of this study. Clades: A - East Asia, E1 - European 1, E2 - European 2, NE and ME - Eastern (Near East and Middle East, respectively). In European 1 (E1) clade, two sides are distinguished: (A) and (C). (PDF) [file pone.0091401.s001.pdf]

Table S1. List of the wild boar and domestic pig mtDNA sequences used in this study. Sequences were downloaded from GenBank or obtained by the authors of this study. Clades: A - East Asia, E1 - European 1, E2 - European 2, NE and ME - Eastern (Near East and Middle East, respectively). In European 1 (E1) clade, two sides are distinguished: (A) and (C).

GenBank accession no.	ID no.	Country of origin	Region (see Fig. 4)	Status	Haplotype (haplotype in this study)	Clade (side)	Reference
JF774408	Kbl020	Kavala, Greece	Balkans	Wild	H15	A	Alexandri et al. 2012
JF774184	Ebr003	Ebros, Greece	Balkans	Wild	H4	E1 (A)	Alexandri et al. 2012
JF774185	Rod012	Rodopi, Greece	Balkans	Wild	H20	E1 (A)	Alexandri et al. 2012
JF774186	ait072	Aitoloakarnania, Greece	Balkans	Wild	H26	E1 (A)	Alexandri et al. 2012
JF774187	drm037	Drama, Greece	Balkans	Wild	H26	E1 (A)	Alexandri et al. 2012
JF774188	Ebr017	Ebros, Greece	Balkans	Wild	H26	E1 (A)	Alexandri et al. 2012
JF774189	trz441	Trikala, Greece	Balkans	Feral	H26	E1 (A)	Alexandri et al. 2012
JF774213	pel350	Pella, Greece	Balkans	Wild	H26	E1 (A)	Alexandri et al. 2012
JF774192	ths128	Thessaloniki, Greece	Balkans	Wild	H27	E1 (A)	Alexandri et al. 2012
JF774193	ioa319	Ioannina, Greece	Balkans	Wild	H28	E1 (A)	Alexandri et al. 2012
JF774198	Ths003	Thessaloniki, Greece	Balkans	Wild	H29	E1 (A)	Alexandri et al. 2012
JF774204	pel355	Pella, Greece	Balkans	Wild	H30	E1 (A)	Alexandri et al. 2012
JF774207	EbrT21	Ebros, Greece	Balkans	Wild	H32	E1 (A)	Alexandri et al. 2012
JF774253	EbrT18	Ebros, Greece	Balkans	Wild	H45	E1 (A)	Alexandri et al. 2012
JF774254	EbrT22	Ebros, Greece	Balkans	Wild	H45	E1 (A)	Alexandri et al. 2012
JF774255	EbrT11	Ebros, Greece	Balkans	Wild	H46	E1 (A)	Alexandri et al. 2012
JF774182	ait440	Aitoloakarnania, Greece	Balkans	Wild	H1	E1 (C)	Alexandri et al. 2012
JF774183	eur061	Evritania, Greece	Balkans	Wild	H1	E1 (C)	Alexandri et al. 2012
JF774219	Ebr020	Ebros, Greece	Balkans	Wild	H1	E1 (C)	Alexandri et al. 2012
JF774229	dra035	Drama, Greece	Balkans	Wild	H1	E1 (C)	Alexandri et al. 2012
JF774230	pel352	Pella, Greece	Balkans	Wild	H1	E1 (C)	Alexandri et al. 2012
JF774231	ark435	Arcadia, Greece	Balkans	Wild	H1	E1 (C)	Alexandri et al. 2012
JF774233	ark439	Arcadia, Greece	Balkans	Wild	H1	E1 (C)	Alexandri et al. 2012
JF774234	dra015	Drama, Greece	Balkans	Wild	H1	E1 (C)	Alexandri et al. 2012
JF774235	krn125	Korinthos, Greece	Balkans	Wild	H1	E1 (C)	Alexandri et al. 2012

JF774236	dra046	Drama, Greece	Balkans	Wild	H1	E1 (C)	Alexandri et al. 2012
JF774237	drm016	Drama, Greece	Balkans	Wild	H1	E1 (C)	Alexandri et al. 2012
JF774238	Rod002	Rodopi, Greece	Balkans	Wild	H1	E1 (C)	Alexandri et al. 2012
JF774239	Rod003	Rodopi, Greece	Balkans	Wild	H1	E1 (C)	Alexandri et al. 2012
JF774240	RodT08	Rodopi, Greece	Balkans	Wild	H1	E1 (C)	Alexandri et al. 2012
JF774284	eur492	Evritania, Greece	Balkans	Wild	H2	E1 (C)	Alexandri et al. 2012
JF774285	eur506	Evritania, Greece	Balkans	Wild	H2	E1 (C)	Alexandri et al. 2012
JF774288	lar223	Larisa, Greece	Balkans	Wild	H2	E1 (C)	Alexandri et al. 2012
JF774289	ait305	Aitoloakarnania, Greece	Balkans	Wild	H2	E1 (C)	Alexandri et al. 2012
JF774290	ait286	Aitoloakarnania, Greece	Balkans	Wild	H2	E1 (C)	Alexandri et al. 2012
JF774291	lar228	Larisa, Greece	Balkans	Wild	H2	E1 (C)	Alexandri et al. 2012
JF774292	lar231	Larisa, Greece	Balkans	Wild	H2	E1 (C)	Alexandri et al. 2012
JF774293	lar234	Larisa, Greece	Balkans	Wild	H2	E1 (C)	Alexandri et al. 2012
JF774294	ait049	Aitoloakarnania, Greece	Balkans	Wild	H2	E1 (C)	Alexandri et al. 2012
JF774295	ioa211	Ioannina, Greece	Balkans	Wild	H2	E1 (C)	Alexandri et al. 2012
JF774296	mag222	Magnisia, Greece	Balkans	Wild	H2	E1 (C)	Alexandri et al. 2012
JF774297	fok116	Fokida, Greece	Balkans	Wild	H2	E1 (C)	Alexandri et al. 2012
JF774298	fok050	Fokida, Greece	Balkans	Wild	H2	E1 (C)	Alexandri et al. 2012
JF774299	tri360	Trikala, Greece	Balkans	Wild	H2	E1 (C)	Alexandri et al. 2012
JF774300	voi236	Voiotia, Greece	Balkans	Wild	H2	E1 (C)	Alexandri et al. 2012
JF774301	voi306	Voiotia, Greece	Balkans	Wild	H2	E1 (C)	Alexandri et al. 2012
JF774302	fok430	Fokida, Greece	Balkans	Wild	H2	E1 (C)	Alexandri et al. 2012
JF774303	fth080	Fthiotida, Greece	Balkans	Wild	H2	E1 (C)	Alexandri et al. 2012
JF774304	fth216	Fthiotida, Greece	Balkans	Wild	H2	E1 (C)	Alexandri et al. 2012
JF774305	eur062	Evritania, Greece	Balkans	Wild	H2	E1 (C)	Alexandri et al. 2012
JF774306	eur307	Evritania, Greece	Balkans	Wild	H2	E1 (C)	Alexandri et al. 2012
JF774307	eur316	Evritania, Greece	Balkans	Wild	H2	E1 (C)	Alexandri et al. 2012
JF774310	krn124	Korinthos, Greece	Balkans	Wild	H2	E1 (C)	Alexandri et al. 2012
JF774311	lar224	Larisa, Greece	Balkans	Wild	H2	E1 (C)	Alexandri et al. 2012
JF774312	ait297	Aitoloakarnania, Greece	Balkans	Wild	H2	E1 (C)	Alexandri et al. 2012
JF774315	mag524	Magnisia, Greece	Balkans	Wild	H2	E1 (C)	Alexandri et al. 2012
JF774316	mag227	Magnisia, Greece	Balkans	Wild	H2	E1 (C)	Alexandri et al. 2012
JF774317	ait278	Aitoloakarnania, Greece	Balkans	Wild	H2	E1 (C)	Alexandri et al. 2012
JF774318	lar230	Larisa, Greece	Balkans	Wild	H2	E1 (C)	Alexandri et al. 2012
JF774319	lar232	Larisa, Greece	Balkans	Wild	H2	E1 (C)	Alexandri et al. 2012
JF774320	ait127	Aitoloakarnania, Greece	Balkans	Wild	H2	E1 (C)	Alexandri et al. 2012
JF774321	ioa509	Ioannina, Greece	Balkans	Wild	H2	E1 (C)	Alexandri et al. 2012
JF774322	lar513	Larisa, Greece	Balkans	Wild	H2	E1 (C)	Alexandri et al. 2012

JF774323	fok064	Fokida, Greece	Balkans	Wild	H2	E1 (C)	Alexandri et al. 2012
JF774324	fok290	Fokida, Greece	Balkans	Wild	H2	E1 (C)	Alexandri et al. 2012
JF774325	voi011	Voiotia, Greece	Balkans	Wild	H2	E1 (C)	Alexandri et al. 2012
JF774326	fth274	Fthiotida, Greece	Balkans	Wild	H2	E1 (C)	Alexandri et al. 2012
JF774327	voi237	Voiotia, Greece	Balkans	Wild	H2	E1 (C)	Alexandri et al. 2012
JF774328	voi422	Voiotia, Greece	Balkans	Wild	H2	E1 (C)	Alexandri et al. 2012
JF774329	fok450	Fokida, Greece	Balkans	Wild	H2	E1 (C)	Alexandri et al. 2012
JF774330	fok082	Fthiotida, Greece	Balkans	Wild	H2	E1 (C)	Alexandri et al. 2012
JF774331	fth217	Fthiotida, Greece	Balkans	Wild	H2	E1 (C)	Alexandri et al. 2012
JF774332	eur088	Evritania, Greece	Balkans	Wild	H2	E1 (C)	Alexandri et al. 2012
JF774333	eur308	Evritania, Greece	Balkans	Wild	H2	E1 (C)	Alexandri et al. 2012
JF774334	eur480	Evritania, Greece	Balkans	Wild	H2	E1 (C)	Alexandri et al. 2012
JF774335	eur312	Evritania, Greece	Balkans	Wild	H2	E1 (C)	Alexandri et al. 2012
JF774336	eur092	Evritania, Greece	Balkans	Wild	H2	E1 (C)	Alexandri et al. 2012
JF774337	fth250	Fthiotida, Greece	Balkans	Wild	H2	E1 (C)	Alexandri et al. 2012
JF774338	fth084	Fthiotida, Greece	Balkans	Wild	H2	E1 (C)	Alexandri et al. 2012
JF774339	fok452	Fokida, Greece	Balkans	Wild	H2	E1 (C)	Alexandri et al. 2012
JF774340	voi423	Voiotia, Greece	Balkans	Wild	H2	E1 (C)	Alexandri et al. 2012
JF774341	voi264	Voiotia, Greece	Balkans	Wild	H2	E1 (C)	Alexandri et al. 2012
JF774342	fth505	Fthiotida, Greece	Balkans	Wild	H2	E1 (C)	Alexandri et al. 2012
JF774343	voi013	Voiotia, Greece	Balkans	Wild	H2	E1 (C)	Alexandri et al. 2012
JF774344	fth255	Fthiotida, Greece	Balkans	Wild	H2	E1 (C)	Alexandri et al. 2012
JF774345	fok115	Fokida, Greece	Balkans	Wild	H2	E1 (C)	Alexandri et al. 2012
JF774346	mag518	Magnisia, Greece	Balkans	Wild	H2	E1 (C)	Alexandri et al. 2012
JF774347	ioa212	Ioannina, Greece	Balkans	Wild	H2	E1 (C)	Alexandri et al. 2012
JF774348	ait500	Aitoloakarnania, Greece	Balkans	Wild	H2	E1 (C)	Alexandri et al. 2012
JF774349	lar233	Larisa, Greece	Balkans	Wild	H2	E1 (C)	Alexandri et al. 2012
JF774350	ait126	Aitoloakarnania, Greece	Balkans	Wild	H2	E1 (C)	Alexandri et al. 2012
JF774351	ait400	Aitoloakarnania, Greece	Balkans	Wild	H2	E1 (C)	Alexandri et al. 2012
JF774352	pel354	Pella, Greece	Balkans	Wild	H2	E1 (C)	Alexandri et al. 2012
JF774363	fth253	Fthiotida, Greece	Balkans	Wild	H2	E1 (C)	Alexandri et al. 2012
JF774364	fth259	Fthiotida, Greece	Balkans	Wild	H2	E1 (C)	Alexandri et al. 2012
JF774365	fth268	Fthiotida, Greece	Balkans	Wild	H2	E1 (C)	Alexandri et al. 2012
JF774366	fth269	Fthiotida, Greece	Balkans	Wild	H2	E1 (C)	Alexandri et al. 2012
JF774367	thp408	Ioannina, Greece	Balkans	Wild	H2	E1 (C)	Alexandri et al. 2012
JF774359	ait109	Aitoloakarnania, Greece	Balkans	Wild	H3	E1 (C)	Alexandri et al. 2012
JF774355	eur310	Evritania, Greece	Balkans	Wild	H5	E1 (C)	Alexandri et al. 2012
JF774358	fok298	Fokida, Greece	Balkans	Wild	H6	E1 (C)	Alexandri et al. 2012

JF774354	fok055	Fokida, Greece	Balkans	Wild	H7	E1 (C)	Alexandri et al. 2012
JF774362	fth254	Fthiotida, Greece	Balkans	Wild	H8	E1 (C)	Alexandri et al. 2012
JF774368	ioa208	Ioannina, Greece	Balkans	Wild	H9	E1 (C)	Alexandri et al. 2012
JF774369	ioa322	Ioannina, Greece	Balkans	Wild	H9	E1 (C)	Alexandri et al. 2012
JF774370	ioa405	Ioannina, Greece	Balkans	Wild	H9	E1 (C)	Alexandri et al. 2012
JF774371	ioa320	Ioannina, Greece	Balkans	Wild	H9	E1 (C)	Alexandri et al. 2012
JF774372	thp407	Ioannina, Greece	Balkans	Wild	H9	E1 (C)	Alexandri et al. 2012
JF774243	dom027	Greece	Balkans	Domestic	HH10	E1 (C)	Alexandri et al. 2012
JF774247	dra001	Drama, Greece	Balkans	Wild	H10	E1 (C)	Alexandri et al. 2012
JF774248	EbrT15	Ebros, Greece	Balkans	Wild	H10	E1 (C)	Alexandri et al. 2012
JF774249	ioa406	Ioannina, Greece	Balkans	Wild	H10	E1 (C)	Alexandri et al. 2012
JF774250	Rod006	Rodopi, Greece	Balkans	Wild	H10	E1 (C)	Alexandri et al. 2012
JF774251	tri357	Trikala, Greece	Balkans	Wild	H10	E1 (C)	Alexandri et al. 2012
JF774256	dra007	Drama, Greece	Balkans	Wild	H10	E1 (C)	Alexandri et al. 2012
JF774257	ioa507	Ioannina, Greece	Balkans	Wild	H10	E1 (C)	Alexandri et al. 2012
JF774258	lar226	Larisa, Greece	Balkans	Wild	H10	E1 (C)	Alexandri et al. 2012
JF774259	tri514	Trikala, Greece	Balkans	Wild	H10	E1 (C)	Alexandri et al. 2012
JF774271	dra005	Drama, Greece	Balkans	Wild	H10	E1 (C)	Alexandri et al. 2012
JF774272	Ebr025	Ebros, Greece	Balkans	Wild	H10	E1 (C)	Alexandri et al. 2012
JF774273	Kbl028	Kavala, Greece	Balkans	Wild	H10	E1 (C)	Alexandri et al. 2012
JF774274	Rod007	Rodopi, Greece	Balkans	Wild	H10	E1 (C)	Alexandri et al. 2012
JF774275	tri359	Trikala, Greece	Balkans	Wild	H10	E1 (C)	Alexandri et al. 2012
JF774276	Blg004	Bulgaria	Balkans	Wild	H10	E1 (C)	Alexandri et al. 2012
JF774278	Blg005	Bulgaria	Balkans	Wild	H10	E1 (C)	Alexandri et al. 2012
JF774374	Kbl026	Kavala, Greece	Balkans	Wild	H10	E1 (C)	Alexandri et al. 2012
JF774376	Kbl023	Kavala, Greece	Balkans	Wild	H11	E1 (C)	Alexandri et al. 2012
JF774260	Ebr008	Ebros, Greece	Balkans	Wild	H12	E1 (C)	Alexandri et al. 2012
JF774261	Ebr018	Ebros, Greece	Balkans	Wild	H12	E1 (C)	Alexandri et al. 2012
JF774262	Ebr022	Ebros, Greece	Balkans	Wild	H12	E1 (C)	Alexandri et al. 2012
JF774263	Hlk002	Halkidiki, Greece	Balkans	Wild	H12	E1 (C)	Alexandri et al. 2012
JF774264	Kbl001	Kavala, Greece	Balkans	Wild	H12	E1 (C)	Alexandri et al. 2012
JF774265	Kbl004	Kavala, Greece	Balkans	Wild	H12	E1 (C)	Alexandri et al. 2012
JF774266	Kbl006	Kavala, Greece	Balkans	Wild	H12	E1 (C)	Alexandri et al. 2012
JF774267	Kbl007	Kavala, Greece	Balkans	Wild	H12	E1 (C)	Alexandri et al. 2012
JF774268	Ths001	Thessaloniki, Greece	Balkans	Wild	H12	E1 (C)	Alexandri et al. 2012
JF774269	Ths002	Thessaloniki, Greece	Balkans	Wild	H12	E1 (C)	Alexandri et al. 2012
JF774270	Ths004	Thessaloniki, Greece	Balkans	Wild	H12	E1 (C)	Alexandri et al. 2012
JF774377	Kbl027	Kavala, Greece	Balkans	Wild	H12	E1 (C)	Alexandri et al. 2012

JF774378	Kbl030	Kavala, Greece	Balkans	Wild	H12	E1 (C)	Alexandri et al. 2012
JF774379	Kbl021	Kavala, Greece	Balkans	Wild	H13	E1 (C)	Alexandri et al. 2012
JF774380	Kbl022	Kavala, Greece	Balkans	Wild	H14	E1 (C)	Alexandri et al. 2012
JF774360	lar225	Larisa, Greece	Balkans	Wild	H16	E1 (C)	Alexandri et al. 2012
JF774361	lar235	Larisa, Greece	Balkans	Wild	H17	E1 (C)	Alexandri et al. 2012
JF774353	mag523	Magnisia, Greece	Balkans	Wild	H18	E1 (C)	Alexandri et al. 2012
JF774373	pel351	Pella, Greece	Balkans	Wild	H19	E1 (C)	Alexandri et al. 2012
JF774375	Blg002	Bulgaria	Balkans	Wild	H24	E1 (C)	Alexandri et al. 2012
JF774356	voi056	Voiotia, Greece	Balkans	Wild	H25	E1 (C)	Alexandri et al. 2012
JF774357	voi242	Voiotia, Greece	Balkans	Wild	H25	E1 (C)	Alexandri et al. 2012
JF774205	Rod001	Rodopi, Greece	Balkans	Wild	H31	E1 (C)	Alexandri et al. 2012
JF774206	Blg003	Bulgaria	Balkans	Wild	H31	E1 (C)	Alexandri et al. 2012
JF774208	Kbl025	Kavala, Greece	Balkans	Wild	H33	E1 (C)	Alexandri et al. 2012
JF774209	Kbl029	Kavala, Greece	Balkans	Wild	H34	E1 (C)	Alexandri et al. 2012
JF774210	pel353	Pella, Greece	Balkans	Wild	H35	E1 (C)	Alexandri et al. 2012
JF774211	ark438	Arcadia, Greece	Balkans	Wild	H36	E1 (C)	Alexandri et al. 2012
JF774212	ark432	Arcadia, Greece	Balkans	Wild	H37	E1 (C)	Alexandri et al. 2012
JF774214	Ebr014	Ebros, Greece	Balkans	Wild	H38	E1 (C)	Alexandri et al. 2012
JF774215	Kbl002	Kavala, Greece	Balkans	Wild	H38	E1 (C)	Alexandri et al. 2012
JF774216	Rod008	Rodopi, Greece	Balkans	Wild	H38	E1 (C)	Alexandri et al. 2012
JF774217	Rod010	Rodopi, Greece	Balkans	Wild	H38	E1 (C)	Alexandri et al. 2012
JF774218	Rod017	Rodopi, Greece	Balkans	Wild	H38	E1 (C)	Alexandri et al. 2012
JF774220	Hlk001	Halkidiki, Greece	Balkans	Wild	H39	E1 (C)	Alexandri et al. 2012
JF774221	Hlk003	Halkidiki, Greece	Balkans	Wild	H39	E1 (C)	Alexandri et al. 2012
JF774222	Hlk004	Halkidiki, Greece	Balkans	Wild	H39	E1 (C)	Alexandri et al. 2012
JF774223	Hlk005	Halkidiki, Greece	Balkans	Wild	H39	E1 (C)	Alexandri et al. 2012
JF774224	Hlk006	Halkidiki, Greece	Balkans	Wild	H39	E1 (C)	Alexandri et al. 2012
JF774225	Hlk007	Halkidiki, Greece	Balkans	Wild	H39	E1 (C)	Alexandri et al. 2012
JF774226	Hlk008	Halkidiki, Greece	Balkans	Wild	H39	E1 (C)	Alexandri et al. 2012
JF774227	Hlk036	Halkidiki, Greece	Balkans	Wild	H40	E1 (C)	Alexandri et al. 2012
JF774228	dra012	Drama, Greece	Balkans	Wild	H41	E1 (C)	Alexandri et al. 2012
JF774232	drm129	Drama, Greece	Balkans	Wild	H42	E1 (C)	Alexandri et al. 2012
JF774241	Kbl012	Kavala, Greece	Balkans	Wild	H43	E1 (C)	Alexandri et al. 2012
JF774244	EbrT05	Ebros, Greece	Balkans	Wild	H44	E1 (C)	Alexandri et al. 2012
JF774245	RodT09	Rodopi, Greece	Balkans	Wild	H44	E1 (C)	Alexandri et al. 2012
JF774246	RodT10	Rodopi, Greece	Balkans	Wild	H44	E1 (C)	Alexandri et al. 2012
JF774252	EbrT01	Ebros, Greece	Balkans	Wild	H45	E1 (C)	Alexandri et al. 2012
JF774277	Rod015	Rodopi, Greece	Balkans	Wild	H47	E1 (C)	Alexandri et al. 2012

JF774279	dra009	Drama, Greece	Balkans	Wild	H48	E1 (C)	Alexandri et al. 2012
JF774280	EbrT27	Ebros, Greece	Balkans	Wild	H49	E1 (C)	Alexandri et al. 2012
JF774281	EbrT03	Ebros, Greece	Balkans	Wild	H50	E1 (C)	Alexandri et al. 2012
JF774282	ioa317	Ioannina, Greece	Balkans	Wild	H51	E1 (C)	Alexandri et al. 2012
JF774283	EbrT04	Ebros, Greece	Balkans	Wild	H52	E1 (C)	Alexandri et al. 2012
JF774286	voi014	Voiotia, Greece	Balkans	Wild	H53	E1 (C)	Alexandri et al. 2012
JF774287	voi445	Voiotia, Greece	Balkans	Wild	H53	E1 (C)	Alexandri et al. 2012
JF774308	ait261	Aitoloakarnania, Greece	Balkans	Wild	H54	E1 (C)	Alexandri et al. 2012
JF774309	fok046	Fokida, Greece	Balkans	Wild	H54	E1 (C)	Alexandri et al. 2012
JF774313	mag229	Magnisia, Greece	Balkans	Wild	H55	E1 (C)	Alexandri et al. 2012
JF774314	voi003	Voiotia, Greece	Balkans	Wild	H56	E1 (C)	Alexandri et al. 2012
JF774242	dom022	Greece	Balkans	Domestic	H57	E1 (C)	Alexandri et al. 2012
JF774381	Sam001	Samos, Greece	Near East	Wild	H21	NE	Alexandri et al. 2012
JF774382	Sam002	Samos, Greece	Near East	Wild	H21	NE	Alexandri et al. 2012
JF774383	Sam006	Samos, Greece	Near East	Wild	H21	NE	Alexandri et al. 2012
JF774384	Sam007	Samos, Greece	Near East	Wild	H22	NE	Alexandri et al. 2012
JF774385	Sam008	Samos, Greece	Near East	Wild	H22	NE	Alexandri et al. 2012
JF774386	Sam010	Samos, Greece	Near East	Wild	H22	NE	Alexandri et al. 2012
JF774387	Sam012	Samos, Greece	Near East	Wild	H22	NE	Alexandri et al. 2012
JF774388	Sam013	Samos, Greece	Near East	Wild	H22	NE	Alexandri et al. 2012
JF774389	Sam014	Samos, Greece	Near East	Wild	H22	NE	Alexandri et al. 2012
JF774390	Sam015	Samos, Greece	Near East	Wild	H22	NE	Alexandri et al. 2012
JF774391	Sam016	Samos, Greece	Near East	Wild	H22	NE	Alexandri et al. 2012
JF774392	Sam004	Samos, Greece	Near East	Wild	H22	NE	Alexandri et al. 2012
JF774393	Sam005	Samos, Greece	Near East	Wild	H23	NE	Alexandri et al. 2012
AY232868	SWB1	Spain	Iberian Peninsula	Wild	H75	E1 (C)	Alves et al 2003
AY232869	SWB2	Spain	Iberian Peninsula	Wild	H75	E1 (C)	Alves et al 2003
AY232870	SWB3	Spain	Iberian Peninsula	Wild	H75	E1 (C)	Alves et al 2003
AY232873	SWB5	Spain	Iberian Peninsula	Wild	H76	E1 (C)	Alves et al 2003
AY232874	SWB7	Spain	Iberian Peninsula	Wild	H77	E1 (C)	Alves et al 2003
AY232892	MG	Hungary	Central Europe	Domestic	H26	E1 (A)	Alves et al. 2003
AY232864	IB23	Iberia	Iberian Peninsula	Domestic	H10	E1 (C)	Alves et al. 2003
AY232846	IB5	Spain	Iberian Peninsula	Domestic	H98	E1 (C)	Alves et al. 2003
AY232847	IB6	Spain	Iberian Peninsula	Domestic	H98	E1 (C)	Alves et al. 2003
AY232848	IB7	Spain	Iberian Peninsula	Domestic	H98	E1 (C)	Alves et al. 2003
DQ152853	MA01	Hungary	Central Europe	Domestic	H28	E1 (A)	Fang & Andersson 2006
DQ379062	MA01-1	Hungary	Central Europe	Domestic	H28	E1 (A)	Fang & Andersson 2006
DQ379063	MA01-2	Hungary	Central Europe	Domestic	H28	E1 (A)	Fang & Andersson 2006
DQ379064	MA01-3	Hungary	Central Europe	Domestic	H28	E1 (A)	Fang & Andersson 2006

DQ152866	PI03-4	Europe	Europe	Domestic	H83	E1 (A)	Fang & Andersson 2006
DQ379229	PI04-1	Europe	Europe	Domestic	H83	E1 (A)	Fang & Andersson 2006
DQ379230	PI04-2	Europe	Europe	Domestic	H83	E1 (A)	Fang & Andersson 2006
DQ379231	PI04-3	Europe	Europe	Domestic	H83	E1 (A)	Fang & Andersson 2006
DQ152847	BB01-3	Germany	Central Europe	Domestic	H10	E1 (C)	Fang & Andersson 2006
DQ379027	BB01-1	Germany	Central Europe	Domestic	H10	E1 (C)	Fang & Andersson 2006
DQ379028	BB01-2	Germany	Central Europe	Domestic	H10	E1 (C)	Fang & Andersson 2006
DQ379077	NI01-1	Spain	Iberian Peninsula	Domestic	H10	E1 (C)	Fang & Andersson 2006
DQ379078	NI01-2	Spain	Iberian Peninsula	Domestic	H10	E1 (C)	Fang & Andersson 2006
DQ379079	NI01-3	Spain	Iberian Peninsula	Domestic	H10	E1 (C)	Fang & Andersson 2006
DQ379253	Sm9	France	Western Europe	Wild	H26	E1 (A)	Fang et al 2006
DQ379245	Sm1	France	Western Europe	Wild	H67	E1 (A)	Fang et al 2006
DQ379255	S2	France	Western Europe	Wild	H82	E1 (A)	Fang et al 2006
DQ379256	S3	France	Western Europe	Wild	H82	E1 (A)	Fang et al 2006
DQ379257	S6	France	Western Europe	Wild	H82	E1 (A)	Fang et al 2006
DQ379258	S7	France	Western Europe	Wild	H82	E1 (A)	Fang et al 2006
DQ379259	S8	France	Western Europe	Wild	H82	E1 (A)	Fang et al 2006
DQ379260	S9	France	Western Europe	Wild	H82	E1 (A)	Fang et al 2006
DQ379261	S10	France	Western Europe	Wild	H82	E1 (A)	Fang et al 2006
DQ379238	96	Belgium	Western Europe	Wild	H83	E1 (A)	Fang et al 2006
DQ379239	105	Belgium	Western Europe	Wild	H83	E1 (A)	Fang et al 2006
DQ379234	78	Belgium	Western Europe	Wild	H85	E1 (A)	Fang et al 2006
DQ379241	114	Belgium	Western Europe	Wild	H10	E1 (C)	Fang et al 2006
DQ379243	mitS2	France	Western Europe	Wild	H67	E1 (C)	Fang et al 2006
DQ379246	Sm2	France	Western Europe	Wild	H67	E1 (C)	Fang et al 2006
DQ379247	Sm3	France	Western Europe	Wild	H67	E1 (C)	Fang et al 2006
DQ379248	Sm4	France	Western Europe	Wild	H67	E1 (C)	Fang et al 2006
DQ379250	Sm6	France	Western Europe	Wild	H67	E1 (C)	Fang et al 2006
DQ379235	81	Belgium	Western Europe	Wild	H75	E1 (C)	Fang et al 2006
DQ379236	83	Belgium	Western Europe	Wild	H75	E1 (C)	Fang et al 2006
DQ379237	89	Belgium	Western Europe	Wild	H75	E1 (C)	Fang et al 2006
DQ379244	mitS3	France	Western Europe	Wild	H84	E1 (C)	Fang et al 2006
AF136564	AWB10	Japan	Far East	Wild	H96	A	Giuffra et al 2000
AF136565	AWB11	Japan	Far East	Wild	H97	A	Giuffra et al 2000
AF136556	EWB2	Poland	Central Europe	Wild	H1	E1 (C)	Giuffra et al 2000
AF136555	EWB1	Poland	Central Europe	Wild	H88	E1 (C)	Giuffra et al 2000
AF136563	EWB3	Italy	Apennine Peninsula	Wild	H62	E2	Giuffra et al 2000
AF535163	GongFin36	Finland	Scandinavia	Wild	H88	E1 (C)	Gongora et al 2003
AF535164	GongFin41	Finland	Scandinavia	Wild	H89	E1 (C)	Gongora et al 2003
AY463072	MANGA	Hungary	Central Europe	Domestic	H28	E1 (A)	Gongora et al. 2004

GQ338953	*Sus barbatus*			Wild outgroup			Gongora et al. 2011
HM026639	WB21	China	Far East	Wild	H73	A	Kim et al. 2011
HM026640	WB22	China	Far East	Wild	H73	A	Kim et al. 2011
HM026611	WB05	France	Western Europe	Wild	H26	E1 (A)	Kim et al. 2011
HM026618	WB12	Slovenia	Central Europe	Wild	H26	E1 (A)	Kim et al. 2011
HM026609	WB03	Belgium	Western Europe	Wild	H28	E1 (A)	Kim et al. 2011
HM026613	WB07	Italy	Apennine Peninsula	Wild	H28	E1 (A)	Kim et al. 2011
HM026610	WB04	Hungary	Central Europe	Wild	H1	E1 (C)	Kim et al. 2011
HM026612	WB06	Poland	Central Europe	Wild	H1	E1 (C)	Kim et al. 2011
HM026614	WB08	Romania	Eastern Europe	Wild	H1	E1 (C)	Kim et al. 2011
HM026608	WB02	Czech Republic	Central Europe	Wild	H67	E1 (C)	Kim et al. 2011
HM026607	WB01	Spain	Iberian Peninsula	Wild	H69	E1 (C)	Kim et al. 2011
HM026615	WB09	Sweden	Scandinavia	Wild	H70	E1 (C)	Kim et al. 2011
HM026616	WB10	Tunisia	North Africa	Wild	H71	E1 (C)	Kim et al. 2011
HM026617	WB11	Morocco	North Africa	Wild	H72	E1 (C)	Kim et al. 2011
AY884626	GL63	Germany	Central Europe	Wild	H26	E1 (A)	Larson et al 2005
AY884665	GL108	Germany	Central Europe	Wild	H26	E1 (A)	Larson et al 2005
AY884669	GL112	Holland	Western Europe	Wild	H26	E1 (A)	Larson et al 2005
AY884664	GL107	Germany	Central Europe	Wild	H28	E1 (A)	Larson et al 2005
AY884815	LeifFrenchWB	France	Western Europe	Wild	H82	E1 (A)	Larson et al 2005
AY884682	GL144	Italy	Apennine Peninsula	Wild	H99	E1 (A)	Larson et al 2005
AY884666	GL109	Germany	Central Europe	Wild	H1	E1 (C)	Larson et al 2005
AY884697	GL221	Spain	Iberian Peninsula	Wild	H1	E1 (C)	Larson et al 2005
AY884670	GL113	Macedonia	Balkans	Wild	H10	E1 (C)	Larson et al 2005
AY884616	GL52	Spain	Iberian Peninsula	Wild	H78	E1 (C)	Larson et al 2005
AY884717	GL245	Italy	Apennine Peninsula	Wild	H59	E2	Larson et al 2005
AY884718	GL246	Italy	Apennine Peninsula	Wild	H59	E2	Larson et al 2005
AY884722	GL250	Italy	Apennine Peninsula	Wild	H59	E2	Larson et al 2005
AY884723	GL251	Italy	Apennine Peninsula	Wild	H59	E2	Larson et al 2005
AY884719	GL247	Italy	Apennine Peninsula	Wild	H62	E2	Larson et al 2005
AY884720	GL248	Italy	Apennine Peninsula	Wild	H62	E2	Larson et al 2005
AY884721	GL249	Italy	Apennine Peninsula	Wild	H62	E2	Larson et al 2005
AY884716	GL244	Italy	Apennine Peninsula	Wild	H80	E2	Larson et al 2005
AY884726	GL270	Armenia	Caucasus	Wild	H81	ME	Larson et al 2005
AY884619	GL55	Turkey	Near East	Wild	H79	NE	Larson et al 2005
DQ872975	GL951	Bulgaria	Balkans	Wild	H82	E1 (A)	Larson et al 2007
DQ872931	GL374	Romania	Eastern Europe	Wild	H86	E1 (A)	Larson et al 2007
DQ872935	GL749	Poland	Central Europe	Wild	H1	E1 (C)	Larson et al 2007
DQ872961	GL799	Romania	Eastern Europe	Wild	H1	E1 (C)	Larson et al 2007
DQ872971	GL946	Hungary	Central Europe	Wild	H1	E1 (C)	Larson et al 2007

DQ872932	GL392	Romania	Eastern Europe	Wild	H68	E1 (C)	Larson et al 2007
DQ872970	GL945	Slovakia	Central Europe	Wild	H68	E1 (C)	Larson et al 2007
DQ872969	GL944	Georgia	Caucasus	Wild	H81	ME	Larson et al 2007
DQ872940	GL754	Turkey	Near East	Wild	H21	NE	Larson et al 2007
DQ872966	GL940	Turkey	Near East	Wild	H87	NE	Larson et al 2007
AY884772	DomLarWhite1	Germany	Central Europe	Domestic	H61	A	Larson et al. 2005
AY884784	AY884784	UK	Western Europe	Domestic	H61	A	Larson et al. 2005
AY884767	›mUKTamwort	UK	Western Europe	Domestic	H95	A	Larson et al. 2005
AY884779	›mFranceCreol	France	Western Europe	Domestic	H95	A	Larson et al. 2005
AY884782	MJ01	Spain	Iberian Peninsula	Domestic	H95	A	Larson et al. 2005
AY884785	DomLarWhite(	UK	Western Europe	Domestic	H95	A	Larson et al. 2005
AY884771	DomMeishan0	UK	Western Europe	Domestic	H101	A	Larson et al. 2005
AY884786	DomLarWhite(	Germany	Central Europe	Domestic	H104	A	Larson et al. 2005
AY884766	.DomPietrain0!	France	Western Europe	Domestic	H26	E1 (A)	Larson et al. 2005
AY884777	›omHampshire	UK	Western Europe	Domestic	H26	E1 (A)	Larson et al. 2005
AY884783	AY884783	UK	Western Europe	Domestic	H26	E1 (A)	Larson et al. 2005
AY884787	DomLandrace1	Germany	Central Europe	Domestic	H26	E1 (A)	Larson et al. 2005
AY884764	nHungaryMan(	Hungary	Central Europe	Domestic	H28	E1 (A)	Larson et al. 2005
AY884773	DomLandrace1	UK	Western Europe	Domestic	H28	E1 (A)	Larson et al. 2005
AY884774	DomLandrace1	UK	Western Europe	Domestic	H28	E1 (A)	Larson et al. 2005
AY884776	DomLandrace(	Denmark	Central Europe	Domestic	H28	E1 (A)	Larson et al. 2005
AY884768	.DomPietrain0	Canary Island	North Africa	Domestic	H83	E1 (A)	Larson et al. 2005
AY884769	.DomPietrain0	Germany	Central Europe	Domestic	H100	E1 (A)	Larson et al. 2005
AY884780	›omBritSaddle	UK	Western Europe	Domestic	H102	E1 (A)	Larson et al. 2005
AY884781	›mGermanyAn	Germany	Central Europe	Domestic	H103	E1 (A)	Larson et al. 2005
AY884765	›omIberiaBlack	Spain	Iberian Peninsula	Domestic	H1	E1 (C)	Larson et al. 2005
AY884770	DomMidWhite(	UK	Western Europe	Domestic	H1	E1 (C)	Larson et al. 2005
AY884778	LDomDuroc03	Germany	Central Europe	Domestic	H10	E1 (C)	Larson et al. 2005
AY884775	›omLandrace0	Finland	Scandinavia	Domestic	H68	E1 (C)	Larson et al. 2005
DQ872981	GL1040	Cyprus	Near East	Domestic	H28	E1 (A)	Larson et al. 2007
AB015094	ropean Wild bo	Italy	Apennine Peninsula	Wild	H59	E2	Okumura et al 2001
AB015095	Wat99ItWB3	Italy	Apennine Peninsula	Wild	H62	E2	Okumura et al 2001
JX894158	Northeast AR	Armenia	Caucasus	Wild	H26	E1 (A)	Ottoni et al. 2013
JX894183	WBTR514	Turkey	Near East	Wild	H26	E1 (A)	Ottoni et al. 2013
JX894151	A298	Armenia	Caucasus	Wild	H106	E1 (A)	Ottoni et al. 2013
JX894152	East AR	Armenia	Caucasus	Wild	H107	E1 (A)	Ottoni et al. 2013
JX894162	Tp1	Turkey	Near East	Wild	H109	E1 (A)	Ottoni et al. 2013
JX894174	WBRO563	Romania	Eastern Europe	Wild	H1	E1 (C)	Ottoni et al. 2013
JX894187	WBUA1267	Ukraine	Eastern Europe	Wild	H1	E1 (C)	Ottoni et al. 2013
JX894188	WBUA1268	Ukraine	Eastern Europe	Wild	H1	E1 (C)	Ottoni et al. 2013

JX894150	AR2	Armenia	Caucasus	Wild	H68	E1 (C)	Ottoni et al. 2013
JX894175	WBTN959	Tunisia	North Africa	Wild	H71	E1 (C)	Ottoni et al. 2013
JX894176	WBTN960	Tunisia	North Africa	Wild	H71	E1 (C)	Ottoni et al. 2013
JX894177	WBTN961	Tunisia	North Africa	Wild	H71	E1 (C)	Ottoni et al. 2013
JX894178	WBTN962	Tunisia	North Africa	Wild	H71	E1 (C)	Ottoni et al. 2013
JX894179	WBTN963	Tunisia	North Africa	Wild	H71	E1 (C)	Ottoni et al. 2013
JX894181	WBTN965	Tunisia	North Africa	Wild	H71	E1 (C)	Ottoni et al. 2013
JX894182	WBTN966	Tunisia	North Africa	Wild	H71	E1 (C)	Ottoni et al. 2013
JX894159	TK107	Turkey	Near East	Wild	H79	NE	Ottoni et al. 2013
JX894160	TK119	Turkey	Near East	Wild	H79	NE	Ottoni et al. 2013
JX894161	TK126	Turkey	Near East	Wild	H79	NE	Ottoni et al. 2013
JX894163	Tp10	Turkey	Near East	Wild	H79	NE	Ottoni et al. 2013
JX894164	Tp11	Turkey	Near East	Wild	H79	NE	Ottoni et al. 2013
JX894165	Tp12	Turkey	Near East	Wild	H79	NE	Ottoni et al. 2013
JX894166	Tp2	Turkey	Near East	Wild	H79	NE	Ottoni et al. 2013
JX894171	Tp7	Turkey	Near East	Wild	H79	NE	Ottoni et al. 2013
JX894172	Tp8	Turkey	Near East	Wild	H79	NE	Ottoni et al. 2013
JX894153	IR 1	Iran	Near East	Wild	H108	NE	Ottoni et al. 2013
JX894154	IR 2	Iran	Near East	Wild	H108	NE	Ottoni et al. 2013
JX894155	IR 4	Iran	Near East	Wild	H108	NE	Ottoni et al. 2013
JX894168	Tp4	Turkey	Near East	Wild	H110	NE	Ottoni et al. 2013
JX894169	Tp5	Turkey	Near East	Wild	H111	NE	Ottoni et al. 2013
JX894170	Tp6	Turkey	Near East	Wild	H112	NE	Ottoni et al. 2013
JX894173	Tp9	Turkey	Near East	Wild	H113	NE	Ottoni et al. 2013
JX894184	WBTR515	Turkey	Near East	Wild	H114	NE	Ottoni et al. 2013
JX894185	WBTR516	Turkey	Near East	Wild	H114	NE	Ottoni et al. 2013
JX894186	WBTR517	Turkey	Near East	Wild	H114	NE	Ottoni et al. 2013
JX894180	WBTN964	Tunisia	North Africa	Wild	H71	NE	Ottoni et al. 2013
AJ314542	SSC1	Italy	Apennine Peninsula	Wild	H1	E1 (C)	Randi et al unp
EU362440	SA6	Italy	Apennine Peninsula	Wild	H61	A	Scandura et al 2008
EU362441	SA7	Italy	Apennine Peninsula	Wild	H61	A	Scandura et al 2008
EU362444	SA10	Italy	Apennine Peninsula	Wild	H61	A	Scandura et al 2008
EU362412	AR20	Italy	Apennine Peninsula	Wild	H26	E1 (A)	Scandura et al 2008
EU362414	AR22	Italy	Apennine Peninsula	Wild	H26	E1 (A)	Scandura et al 2008
EU362415	AR23	Italy	Apennine Peninsula	Wild	H26	E1 (A)	Scandura et al 2008
EU362449	FI33	Italy	Apennine Peninsula	Wild	H26	E1 (A)	Scandura et al 2008
EU362450	FI34	Italy	Apennine Peninsula	Wild	H26	E1 (A)	Scandura et al 2008
EU362452	FI37	Italy	Apennine Peninsula	Wild	H26	E1 (A)	Scandura et al 2008
EU362468	SR10	Italy	Apennine Peninsula	Wild	H26	E1 (A)	Scandura et al 2008
EU362475	SR20	Italy	Apennine Peninsula	Wild	H26	E1 (A)	Scandura et al 2008

EU362487	SS4	Italy	Apennine Peninsula	Wild	H26	E1 (A)	Scandura et al 2008
EU362488	SS16	Italy	Apennine Peninsula	Wild	H26	E1 (A)	Scandura et al 2008
EU362489	SS33	Italy	Apennine Peninsula	Wild	H26	E1 (A)	Scandura et al 2008
EU362496	SS48	Italy	Apennine Peninsula	Wild	H26	E1 (A)	Scandura et al 2008
EU362521	FR11	France	Western Europe	Wild	H26	E1 (A)	Scandura et al 2008
EU362410	AR17	Italy	Apennine Peninsula	Wild	H28	E1 (A)	Scandura et al 2008
EU362418	FO2	Italy	Apennine Peninsula	Wild	H28	E1 (A)	Scandura et al 2008
EU362419	FO3	Italy	Apennine Peninsula	Wild	H28	E1 (A)	Scandura et al 2008
EU362420	FO4	Italy	Apennine Peninsula	Wild	H28	E1 (A)	Scandura et al 2008
EU362426	FO10	Italy	Apennine Peninsula	Wild	H28	E1 (A)	Scandura et al 2008
EU362428	SI2	Italy	Apennine Peninsula	Wild	H28	E1 (A)	Scandura et al 2008
EU362429	SI3	Italy	Apennine Peninsula	Wild	H28	E1 (A)	Scandura et al 2008
EU362430	SI4	Italy	Apennine Peninsula	Wild	H28	E1 (A)	Scandura et al 2008
EU362431	SI5	Italy	Apennine Peninsula	Wild	H28	E1 (A)	Scandura et al 2008
EU362432	SI6	Italy	Apennine Peninsula	Wild	H28	E1 (A)	Scandura et al 2008
EU362433	SI7	Italy	Apennine Peninsula	Wild	H28	E1 (A)	Scandura et al 2008
EU362434	SI9	Italy	Apennine Peninsula	Wild	H28	E1 (A)	Scandura et al 2008
EU362436	SA2	Italy	Apennine Peninsula	Wild	H28	E1 (A)	Scandura et al 2008
EU362445	FI27	Italy	Apennine Peninsula	Wild	H28	E1 (A)	Scandura et al 2008
EU362446	FI29	Italy	Apennine Peninsula	Wild	H28	E1 (A)	Scandura et al 2008
EU362447	FI30	Italy	Apennine Peninsula	Wild	H28	E1 (A)	Scandura et al 2008
EU362534	AS13	Austria	Central Europe	Wild	H28	E1 (A)	Scandura et al 2008
EU362435	SA1	Italy	Apennine Peninsula	Wild	H60	E1 (A)	Scandura et al 2008
EU362498	GO1	Italy	Apennine Peninsula	Wild	H65	E1 (A)	Scandura et al 2008
EU362499	GO4	Italy	Apennine Peninsula	Wild	H65	E1 (A)	Scandura et al 2008
EU362500	GO5	Italy	Apennine Peninsula	Wild	H65	E1 (A)	Scandura et al 2008
EU362501	GO6	Italy	Apennine Peninsula	Wild	H65	E1 (A)	Scandura et al 2008
EU362502	GO11	Italy	Apennine Peninsula	Wild	H65	E1 (A)	Scandura et al 2008
EU362503	GO13	Italy	Apennine Peninsula	Wild	H65	E1 (A)	Scandura et al 2008
EU362504	GO15	Italy	Apennine Peninsula	Wild	H65	E1 (A)	Scandura et al 2008
EU362505	GO18	Italy	Apennine Peninsula	Wild	H65	E1 (A)	Scandura et al 2008
EU362506	GO19	Italy	Apennine Peninsula	Wild	H65	E1 (A)	Scandura et al 2008
EU362526	AS1	Austria	Central Europe	Wild	H66	E1 (A)	Scandura et al 2008
EU362529	AS5	Austria	Central Europe	Wild	H66	E1 (A)	Scandura et al 2008
EU362530	AS6	Austria	Central Europe	Wild	H66	E1 (A)	Scandura et al 2008
EU362531	AS10	Austria	Central Europe	Wild	H66	E1 (A)	Scandura et al 2008
EU362535	AS14	Austria	Central Europe	Wild	H66	E1 (A)	Scandura et al 2008
EU362490	SS36	Italy	Apennine Peninsula	Wild	H1	E1 (C)	Scandura et al 2008
EU362491	SS37	Italy	Apennine Peninsula	Wild	H1	E1 (C)	Scandura et al 2008
EU362492	SS38	Italy	Apennine Peninsula	Wild	H1	E1 (C)	Scandura et al 2008

EU362527	AS3	Austria	Central Europe	Wild	H1	E1 (C)	Scandura et al 2008
EU362536	PO8	Poland	Central Europe	Wild	H1	E1 (C)	Scandura et al 2008
EU362538	PO12	Poland	Central Europe	Wild	H1	E1 (C)	Scandura et al 2008
EU362542	PO25	Poland	Central Europe	Wild	H1	E1 (C)	Scandura et al 2008
EU362543	PO27	Poland	Central Europe	Wild	H1	E1 (C)	Scandura et al 2008
EU362544	UN1	Hungary	Central Europe	Wild	H1	E1 (C)	Scandura et al 2008
EU362545	UN2	Hungary	Central Europe	Wild	H1	E1 (C)	Scandura et al 2008
EU362546	UN6	Hungary	Central Europe	Wild	H1	E1 (C)	Scandura et al 2008
EU362547	UN9	Hungary	Central Europe	Wild	H1	E1 (C)	Scandura et al 2008
EU362548	UN10	Hungary	Central Europe	Wild	H1	E1 (C)	Scandura et al 2008
EU362549	UN15	Hungary	Central Europe	Wild	H1	E1 (C)	Scandura et al 2008
EU362550	UN17	Hungary	Central Europe	Wild	H1	E1 (C)	Scandura et al 2008
EU362551	UN18	Hungary	Central Europe	Wild	H1	E1 (C)	Scandura et al 2008
EU362507	SP2	Spain	Iberian Peninsula	Wild	H10	E1 (C)	Scandura et al 2008
EU362509	SP6	Spain	Iberian Peninsula	Wild	H10	E1 (C)	Scandura et al 2008
EU362510	SP8	Spain	Iberian Peninsula	Wild	H10	E1 (C)	Scandura et al 2008
EU362512	SP10	Spain	Iberian Peninsula	Wild	H10	E1 (C)	Scandura et al 2008
EU362514	SP13	Spain	Iberian Peninsula	Wild	H10	E1 (C)	Scandura et al 2008
EU362515	SP15	Spain	Iberian Peninsula	Wild	H10	E1 (C)	Scandura et al 2008
EU362409	AR16	Italy	Apennine Peninsula	Wild	H58	E1 (C)	Scandura et al 2008
EU362411	AR18	Italy	Apennine Peninsula	Wild	H58	E1 (C)	Scandura et al 2008
EU362417	FO1	Italy	Apennine Peninsula	Wild	H58	E1 (C)	Scandura et al 2008
EU362422	FO6	Italy	Apennine Peninsula	Wild	H58	E1 (C)	Scandura et al 2008
EU362423	FO7	Italy	Apennine Peninsula	Wild	H58	E1 (C)	Scandura et al 2008
EU362425	FO9	Italy	Apennine Peninsula	Wild	H58	E1 (C)	Scandura et al 2008
EU362438	SA4	Italy	Apennine Peninsula	Wild	H58	E1 (C)	Scandura et al 2008
EU362442	SA8	Italy	Apennine Peninsula	Wild	H58	E1 (C)	Scandura et al 2008
EU362451	FI36	Italy	Apennine Peninsula	Wild	H58	E1 (C)	Scandura et al 2008
EU362454	FI47	Italy	Apennine Peninsula	Wild	H58	E1 (C)	Scandura et al 2008
EU362461	MR8	Italy	Apennine Peninsula	Wild	H58	E1 (C)	Scandura et al 2008
EU362463	MR11	Italy	Apennine Peninsula	Wild	H58	E1 (C)	Scandura et al 2008
EU362464	MR12	Italy	Apennine Peninsula	Wild	H58	E1 (C)	Scandura et al 2008
EU362537	PO11	Poland	Central Europe	Wild	H67	E1 (C)	Scandura et al 2008
EU362539	PO14	Poland	Central Europe	Wild	H67	E1 (C)	Scandura et al 2008
EU362540	PO16	Poland	Central Europe	Wild	H67	E1 (C)	Scandura et al 2008
EU362541	PO24	Poland	Central Europe	Wild	H67	E1 (C)	Scandura et al 2008
EU362552	UN24	Hungary	Central Europe	Wild	H68	E1 (C)	Scandura et al 2008
EU362413	AR21	Italy	Apennine Peninsula	Wild	H59	E2	Scandura et al 2008
EU362416	AR24	Italy	Apennine Peninsula	Wild	H59	E2	Scandura et al 2008
EU362455	MR1	Italy	Apennine Peninsula	Wild	H59	E2	Scandura et al 2008

EU362456	MR2	Italy	Apennine Peninsula	Wild	H59	E2	Scandura et al 2008
EU362459	MR5	Italy	Apennine Peninsula	Wild	H59	E2	Scandura et al 2008
EU362460	MR6	Italy	Apennine Peninsula	Wild	H59	E2	Scandura et al 2008
EU362469	SR11	Italy	Apennine Peninsula	Wild	H59	E2	Scandura et al 2008
EU362476	CP1	Italy	Apennine Peninsula	Wild	H59	E2	Scandura et al 2008
EU362443	SA9	Italy	Apennine Peninsula	Wild	H62	E2	Scandura et al 2008
EU362453	FI40	Italy	Apennine Peninsula	Wild	H62	E2	Scandura et al 2008
EU362478	CP8	Italy	Apennine Peninsula	Wild	H62	E2	Scandura et al 2008
EU362479	CP10	Italy	Apennine Peninsula	Wild	H62	E2	Scandura et al 2008
EU362480	CP13	Italy	Apennine Peninsula	Wild	H62	E2	Scandura et al 2008
EU362481	CP14	Italy	Apennine Peninsula	Wild	H62	E2	Scandura et al 2008
EU362482	CP15	Italy	Apennine Peninsula	Wild	H62	E2	Scandura et al 2008
EU362483	CP16	Italy	Apennine Peninsula	Wild	H62	E2	Scandura et al 2008
EU362484	CP20	Italy	Apennine Peninsula	Wild	H63	E2	Scandura et al 2008
EU362486	SS1	Italy	Apennine Peninsula	Wild	H64	E2	Scandura et al 2008
EU362559	CS34	Italy	Apennine Peninsula	Domestic	H26	E1 (A)	Scandura et al. 2008
EU362560	CS36	Italy	Apennine Peninsula	Domestic	H26	E1 (A)	Scandura et al. 2008
EU362562	CS38	Italy	Apennine Peninsula	Domestic	H26	E1 (A)	Scandura et al. 2008
EU362563	CS39	Italy	Apennine Peninsula	Domestic	H26	E1 (A)	Scandura et al. 2008
EU362564	CS40	Italy	Apennine Peninsula	Domestic	H26	E1 (A)	Scandura et al. 2008
EU362565	CS43	Italy	Apennine Peninsula	Domestic	H26	E1 (A)	Scandura et al. 2008
EU362561	CS37	Italy	Apennine Peninsula	Domestic	H10	E1 (C)	Scandura et al. 2008
EU362557	MS26	Italy	Apennine Peninsula	Domestic	H105	E1 (C)	Scandura et al. 2008
EU362558	MS28	Italy	Apennine Peninsula	Domestic	H105	E1 (C)	Scandura et al. 2008
JN031503	PWB39-SW8	Portugal	Iberian Peninsula	Wild	H95	A	van Asch et al. 2012
JN031504	PWB40-SW8	Portugal	Iberian Peninsula	Wild	H95	A	van Asch et al. 2012
JN031505	PWB41-SW8	Portugal	Iberian Peninsula	Wild	H95	A	van Asch et al. 2012
JN031506	PWB43-SW8	Portugal	Iberian Peninsula	Wild	H95	A	van Asch et al. 2012
JN031507	PWB44-SW8	Portugal	Iberian Peninsula	Wild	H95	A	van Asch et al. 2012
JN031508	PWB45-SW8	Portugal	Iberian Peninsula	Wild	H95	A	van Asch et al. 2012
JN031509	PWB46-SW8	Portugal	Iberian Peninsula	Wild	H95	A	van Asch et al. 2012
JN031510	PWB47-SW8	Portugal	Iberian Peninsula	Wild	H95	A	van Asch et al. 2012
JN031511	PWB48-SW8	Portugal	Iberian Peninsula	Wild	H95	A	van Asch et al. 2012
JN031512	PWB51-SW8	Portugal	Iberian Peninsula	Wild	H95	A	van Asch et al. 2012
JN031356	PWB2-S8	Portugal	Iberian Peninsula	Wild	H91	E1 (A)	van Asch et al. 2012
JN031357	PWB3-S8	Portugal	Iberian Peninsula	Wild	H91	E1 (A)	van Asch et al. 2012
JN031358	PWB4-S8	Portugal	Iberian Peninsula	Wild	H91	E1 (A)	van Asch et al. 2012
JN031156	PWB109-S2	Portugal	Iberian Peninsula	Wild	H1	E1 (C)	van Asch et al. 2012
JN031157	PWB111-S2	Portugal	Iberian Peninsula	Wild	H1	E1 (C)	van Asch et al. 2012
JN031158	PWB114-S2	Portugal	Iberian Peninsula	Wild	H1	E1 (C)	van Asch et al. 2012

JN031159	PWB116-S2	Portugal	Iberian Peninsula	Wild	H1	E1 (C)	van Asch et al. 2012
JN031411	PWB1-SW1	Portugal	Iberian Peninsula	Wild	H1	E1 (C)	van Asch et al. 2012
JN031412	PWB7-SW1	Portugal	Iberian Peninsula	Wild	H1	E1 (C)	van Asch et al. 2012
JN031413	PWB8-SW1	Portugal	Iberian Peninsula	Wild	H1	E1 (C)	van Asch et al. 2012
JN031414	PWB9-SW1	Portugal	Iberian Peninsula	Wild	H1	E1 (C)	van Asch et al. 2012
JN031415	PWB10-SW1	Portugal	Iberian Peninsula	Wild	H1	E1 (C)	van Asch et al. 2012
JN031416	PWB11-SW1	Portugal	Iberian Peninsula	Wild	H1	E1 (C)	van Asch et al. 2012
JN031417	PWB12-SW1	Portugal	Iberian Peninsula	Wild	H1	E1 (C)	van Asch et al. 2012
JN031418	PWB13-SW1	Portugal	Iberian Peninsula	Wild	H1	E1 (C)	van Asch et al. 2012
JN031419	PWB14-SW1	Portugal	Iberian Peninsula	Wild	H1	E1 (C)	van Asch et al. 2012
JN031420	PWB15-SW1	Portugal	Iberian Peninsula	Wild	H1	E1 (C)	van Asch et al. 2012
JN031421	PWB16-SW1	Portugal	Iberian Peninsula	Wild	H1	E1 (C)	van Asch et al. 2012
JN031422	PWB17-SW1	Portugal	Iberian Peninsula	Wild	H1	E1 (C)	van Asch et al. 2012
JN031423	PWB18-SW1	Portugal	Iberian Peninsula	Wild	H1	E1 (C)	van Asch et al. 2012
JN031424	PWB19-SW1	Portugal	Iberian Peninsula	Wild	H1	E1 (C)	van Asch et al. 2012
JN031425	PWB34-SW1	Portugal	Iberian Peninsula	Wild	H1	E1 (C)	van Asch et al. 2012
JN031426	PWB35-SW1	Portugal	Iberian Peninsula	Wild	H1	E1 (C)	van Asch et al. 2012
JN031427	PWB36-SW1	Portugal	Iberian Peninsula	Wild	H1	E1 (C)	van Asch et al. 2012
JN031428	PWB37-SW1	Portugal	Iberian Peninsula	Wild	H1	E1 (C)	van Asch et al. 2012
JN031429	PWB38-SW1	Portugal	Iberian Peninsula	Wild	H1	E1 (C)	van Asch et al. 2012
JN031430	PWB75-SW1	Portugal	Iberian Peninsula	Wild	H1	E1 (C)	van Asch et al. 2012
JN031431	PWB76-SW1	Portugal	Iberian Peninsula	Wild	H1	E1 (C)	van Asch et al. 2012
JN031432	PWB77-SW1	Portugal	Iberian Peninsula	Wild	H1	E1 (C)	van Asch et al. 2012
JN031433	PWB78-SW1	Portugal	Iberian Peninsula	Wild	H1	E1 (C)	van Asch et al. 2012
JN031434	PWB79-SW1	Portugal	Iberian Peninsula	Wild	H1	E1 (C)	van Asch et al. 2012
JN031435	PWB80-SW1	Portugal	Iberian Peninsula	Wild	H1	E1 (C)	van Asch et al. 2012
JN031436	PWB81-SW1	Portugal	Iberian Peninsula	Wild	H1	E1 (C)	van Asch et al. 2012
JN031437	PWB82-SW1	Portugal	Iberian Peninsula	Wild	H1	E1 (C)	van Asch et al. 2012
JN031438	PWB83-SW1	Portugal	Iberian Peninsula	Wild	H1	E1 (C)	van Asch et al. 2012
JN031439	PWB84-SW1	Portugal	Iberian Peninsula	Wild	H1	E1 (C)	van Asch et al. 2012
JN031440	PWB85-SW1	Portugal	Iberian Peninsula	Wild	H1	E1 (C)	van Asch et al. 2012
JN031441	PWB86-SW1	Portugal	Iberian Peninsula	Wild	H1	E1 (C)	van Asch et al. 2012
JN031442	PWB87-SW1	Portugal	Iberian Peninsula	Wild	H1	E1 (C)	van Asch et al. 2012
JN031443	PWB88-SW1	Portugal	Iberian Peninsula	Wild	H1	E1 (C)	van Asch et al. 2012
JN031497	PWB42-SW6	Portugal	Iberian Peninsula	Wild	H1	E1 (C)	van Asch et al. 2012
JN031498	PWB49-SW6	Portugal	Iberian Peninsula	Wild	H1	E1 (C)	van Asch et al. 2012
JN031499	PWB50-SW6	Portugal	Iberian Peninsula	Wild	H1	E1 (C)	van Asch et al. 2012
JN031500	PWB52-SW6	Portugal	Iberian Peninsula	Wild	H1	E1 (C)	van Asch et al. 2012
JN031283	PWB117-S6	Portugal	Iberian Peninsula	Wild	H10	E1 (C)	van Asch et al. 2012
JN031494	PWB90-SW4	Portugal	Iberian Peninsula	Wild	H10	E1 (C)	van Asch et al. 2012

JN031495	PWB108-SW4	Portugal	Iberian Peninsula	Wild	H10	E1 (C)	van Asch et al. 2012
JN031462	PWB21-SW3	Portugal	Iberian Peninsula	Wild	H77	E1 (C)	van Asch et al. 2012
JN031463	PWB22-SW3	Portugal	Iberian Peninsula	Wild	H77	E1 (C)	van Asch et al. 2012
JN031464	PWB23-SW3	Portugal	Iberian Peninsula	Wild	H77	E1 (C)	van Asch et al. 2012
JN031465	PWB24-SW3	Portugal	Iberian Peninsula	Wild	H77	E1 (C)	van Asch et al. 2012
JN031466	PWB25-SW3	Portugal	Iberian Peninsula	Wild	H77	E1 (C)	van Asch et al. 2012
JN031467	PWB26-SW3	Portugal	Iberian Peninsula	Wild	H77	E1 (C)	van Asch et al. 2012
JN031468	PWB27-SW3	Portugal	Iberian Peninsula	Wild	H77	E1 (C)	van Asch et al. 2012
JN031469	PWB28-SW3	Portugal	Iberian Peninsula	Wild	H77	E1 (C)	van Asch et al. 2012
JN031470	PWB29-SW3	Portugal	Iberian Peninsula	Wild	H77	E1 (C)	van Asch et al. 2012
JN031471	PWB30-SW3	Portugal	Iberian Peninsula	Wild	H77	E1 (C)	van Asch et al. 2012
JN031472	PWB31-SW3	Portugal	Iberian Peninsula	Wild	H77	E1 (C)	van Asch et al. 2012
JN031473	PWB32-SW3	Portugal	Iberian Peninsula	Wild	H77	E1 (C)	van Asch et al. 2012
JN031474	PWB33-SW3	Portugal	Iberian Peninsula	Wild	H77	E1 (C)	van Asch et al. 2012
JN031475	PWB62-SW3	Portugal	Iberian Peninsula	Wild	H77	E1 (C)	van Asch et al. 2012
JN031476	PWB63-SW3	Portugal	Iberian Peninsula	Wild	H77	E1 (C)	van Asch et al. 2012
JN031477	PWB64-SW3	Portugal	Iberian Peninsula	Wild	H77	E1 (C)	van Asch et al. 2012
JN031478	PWB65-SW3	Portugal	Iberian Peninsula	Wild	H77	E1 (C)	van Asch et al. 2012
JN031479	PWB66-SW3	Portugal	Iberian Peninsula	Wild	H77	E1 (C)	van Asch et al. 2012
JN031480	PWB67-SW3	Portugal	Iberian Peninsula	Wild	H77	E1 (C)	van Asch et al. 2012
JN031481	PWB68-SW3	Portugal	Iberian Peninsula	Wild	H77	E1 (C)	van Asch et al. 2012
JN031482	PWB69-SW3	Portugal	Iberian Peninsula	Wild	H77	E1 (C)	van Asch et al. 2012
JN031483	PWB70-SW3	Portugal	Iberian Peninsula	Wild	H77	E1 (C)	van Asch et al. 2012
JN031484	PWB71-SW3	Portugal	Iberian Peninsula	Wild	H77	E1 (C)	van Asch et al. 2012
JN031485	PWB72-SW3	Portugal	Iberian Peninsula	Wild	H77	E1 (C)	van Asch et al. 2012
JN031486	PWB73-SW3	Portugal	Iberian Peninsula	Wild	H77	E1 (C)	van Asch et al. 2012
JN031487	PWB74-SW3	Portugal	Iberian Peninsula	Wild	H77	E1 (C)	van Asch et al. 2012
JN031488	PWB89-SW3	Portugal	Iberian Peninsula	Wild	H77	E1 (C)	van Asch et al. 2012
JN031489	PWB107-SW3	Portugal	Iberian Peninsula	Wild	H77	E1 (C)	van Asch et al. 2012
JN031490	PWB110-SW3	Portugal	Iberian Peninsula	Wild	H77	E1 (C)	van Asch et al. 2012
JN031491	PWB112-SW3	Portugal	Iberian Peninsula	Wild	H77	E1 (C)	van Asch et al. 2012
JN031492	PWB113-SW3	Portugal	Iberian Peninsula	Wild	H77	E1 (C)	van Asch et al. 2012
JN031493	PWB115-SW3	Portugal	Iberian Peninsula	Wild	H77	E1 (C)	van Asch et al. 2012
JN031329	PWB57-S7	Portugal	Iberian Peninsula	Wild	H90	E1 (C)	van Asch et al. 2012
JN031330	PWB58-S7	Portugal	Iberian Peninsula	Wild	H90	E1 (C)	van Asch et al. 2012
JN031331	PWB59-S7	Portugal	Iberian Peninsula	Wild	H90	E1 (C)	van Asch et al. 2012
JN031332	PWB60-S7	Portugal	Iberian Peninsula	Wild	H90	E1 (C)	van Asch et al. 2012
JN031333	PWB61-S7	Portugal	Iberian Peninsula	Wild	H90	E1 (C)	van Asch et al. 2012
JN031334	PWB102-S7	Portugal	Iberian Peninsula	Wild	H90	E1 (C)	van Asch et al. 2012
JN031335	PWB105-S7	Portugal	Iberian Peninsula	Wild	H90	E1 (C)	van Asch et al. 2012

JN031444	PWB5-SW2	Portugal	Iberian Peninsula	Wild	H92	E1 (C)	van Asch et al. 2012
JN031445	PWB6-SW2	Portugal	Iberian Peninsula	Wild	H92	E1 (C)	van Asch et al. 2012
JN031446	PWB20-SW2	Portugal	Iberian Peninsula	Wild	H92	E1 (C)	van Asch et al. 2012
JN031447	PWB53-SW2	Portugal	Iberian Peninsula	Wild	H92	E1 (C)	van Asch et al. 2012
JN031448	PWB56-SW2	Portugal	Iberian Peninsula	Wild	H92	E1 (C)	van Asch et al. 2012
JN031449	PWB91-SW2	Portugal	Iberian Peninsula	Wild	H92	E1 (C)	van Asch et al. 2012
JN031450	PWB92-SW2	Portugal	Iberian Peninsula	Wild	H92	E1 (C)	van Asch et al. 2012
JN031451	PWB93-SW2	Portugal	Iberian Peninsula	Wild	H92	E1 (C)	van Asch et al. 2012
JN031452	PWB94-SW2	Portugal	Iberian Peninsula	Wild	H92	E1 (C)	van Asch et al. 2012
JN031453	PWB95-SW2	Portugal	Iberian Peninsula	Wild	H92	E1 (C)	van Asch et al. 2012
JN031454	PWB96-SW2	Portugal	Iberian Peninsula	Wild	H92	E1 (C)	van Asch et al. 2012
JN031455	PWB97-SW2	Portugal	Iberian Peninsula	Wild	H92	E1 (C)	van Asch et al. 2012
JN031456	PWB98-SW2	Portugal	Iberian Peninsula	Wild	H92	E1 (C)	van Asch et al. 2012
JN031457	PWB99-SW2	Portugal	Iberian Peninsula	Wild	H92	E1 (C)	van Asch et al. 2012
JN031458	PWB100-SW2	Portugal	Iberian Peninsula	Wild	H92	E1 (C)	van Asch et al. 2012
JN031459	PWB103-SW2	Portugal	Iberian Peninsula	Wild	H92	E1 (C)	van Asch et al. 2012
JN031460	PWB104-SW2	Portugal	Iberian Peninsula	Wild	H92	E1 (C)	van Asch et al. 2012
JN031461	PWB106-SW2	Portugal	Iberian Peninsula	Wild	H92	E1 (C)	van Asch et al. 2012
JN031496	PWB101-SW5	Portugal	Iberian Peninsula	Wild	H93	E1 (C)	van Asch et al. 2012
JN031501	PWB54-SW7	Portugal	Iberian Peninsula	Wild	H94	E1 (C)	van Asch et al. 2012
JN031502	PWB55-SW7	Portugal	Iberian Peninsula	Wild	H94	E1 (C)	van Asch et al. 2012
AJ314540	*Sus barbatus*			Wild outgroup			Wu et al. 2007
	RUS451	Russia	Eastern Europe (this study)	Wild	H97(16)	A	this study
	BEL097	Belarus	Eastern Europe (this study)	Wild	H26 (3)	E1 (A)	this study
	BEL118	Belarus	Eastern Europe (this study)	Wild	H26 (3)	E1 (A)	this study
	BEL120	Belarus	Eastern Europe (this study)	Wild	H26 (3)	E1 (A)	this study
	POL411	Poland	Central Europe (this study)	Wild	H26 (3)	E1 (A)	this study
	POL412	Poland	Central Europe (this study)	Wild	H26 (3)	E1 (A)	this study
	POL414	Poland	Central Europe (this study)	Wild	H26 (3)	E1 (A)	this study
	POL424	Poland	Central Europe (this study)	Wild	H26 (3)	E1 (A)	this study
	POL426	Poland	Central Europe (this study)	Wild	H26 (3)	E1 (A)	this study
	POL485	Poland	Central Europe (this study)	Wild	H26 (3)	E1 (A)	this study
	POL491	Poland	Central Europe (this study)	Wild	H26 (3)	E1 (A)	this study
	RUS04	Russia	Eastern Europe (this study)	Wild	H26 (3)	E1 (A)	this study
	UA05	Ukraine	Eastern Europe (this study)	Wild	H26 (3)	E1 (A)	this study
	RUS449	Russia	Eastern Europe (this study)	Wild	H120 (15)	E1 (C)	this study
	BEL016	Belarus	Eastern Europe (this study)	Wild	H1 (1)	E1 (C)	this study
	BEL046	Belarus	Eastern Europe (this study)	Wild	H1 (1)	E1 (C)	this study
	BEL047	Belarus	Eastern Europe (this study)	Wild	H1 (1)	E1 (C)	this study
	BEL052	Belarus	Eastern Europe (this study)	Wild	H1 (1)	E1 (C)	this study

BEL058	Belarus	Eastern Europe (this study)	Wild	H1 (1)	E1 (C)	this study
BEL062	Belarus	Eastern Europe (this study)	Wild	H1 (1)	E1 (C)	this study
BEL064	Belarus	Eastern Europe (this study)	Wild	H1 (1)	E1 (C)	this study
BEL070	Belarus	Eastern Europe (this study)	Wild	H1 (1)	E1 (C)	this study
BEL073	Belarus	Eastern Europe (this study)	Wild	H1 (1)	E1 (C)	this study
BEL075	Belarus	Eastern Europe (this study)	Wild	H1 (1)	E1 (C)	this study
BEL079	Belarus	Eastern Europe (this study)	Wild	H1 (1)	E1 (C)	this study
BEL087	Belarus	Eastern Europe (this study)	Wild	H1 (1)	E1 (C)	this study
BEL088	Belarus	Eastern Europe (this study)	Wild	H1 (1)	E1 (C)	this study
BEL089	Belarus	Eastern Europe (this study)	Wild	H1 (1)	E1 (C)	this study
BEL100	Belarus	Eastern Europe (this study)	Wild	H1 (1)	E1 (C)	this study
BEL104	Belarus	Eastern Europe (this study)	Wild	H1 (1)	E1 (C)	this study
BEL113	Belarus	Eastern Europe (this study)	Wild	H1 (1)	E1 (C)	this study
BEL116	Belarus	Eastern Europe (this study)	Wild	H1 (1)	E1 (C)	this study
BEL122	Belarus	Eastern Europe (this study)	Wild	H1 (1)	E1 (C)	this study
BEL123	Belarus	Eastern Europe (this study)	Wild	H1 (1)	E1 (C)	this study
BEL124	Belarus	Eastern Europe (this study)	Wild	H1 (1)	E1 (C)	this study
BEL125	Belarus	Eastern Europe (this study)	Wild	H1 (1)	E1 (C)	this study
BEL130	Belarus	Eastern Europe (this study)	Wild	H1 (1)	E1 (C)	this study
BEL131	Belarus	Eastern Europe (this study)	Wild	H1 (1)	E1 (C)	this study
BEL135	Belarus	Eastern Europe (this study)	Wild	H1 (1)	E1 (C)	this study
BEL139	Belarus	Eastern Europe (this study)	Wild	H1 (1)	E1 (C)	this study
BEL141	Belarus	Eastern Europe (this study)	Wild	H1 (1)	E1 (C)	this study
BEL142	Belarus	Eastern Europe (this study)	Wild	H1 (1)	E1 (C)	this study
BEL145	Belarus	Eastern Europe (this study)	Wild	H1 (1)	E1 (C)	this study
BEL148	Belarus	Eastern Europe (this study)	Wild	H1 (1)	E1 (C)	this study
BEL151	Belarus	Eastern Europe (this study)	Wild	H1 (1)	E1 (C)	this study
BEL156	Belarus	Eastern Europe (this study)	Wild	H1 (1)	E1 (C)	this study
BEL163	Belarus	Eastern Europe (this study)	Wild	H1 (1)	E1 (C)	this study
BEL165	Belarus	Eastern Europe (this study)	Wild	H1 (1)	E1 (C)	this study
HU10	Hungary	Central Europe (this study)	Wild	H1 (1)	E1 (C)	this study
HU14	Hungary	Central Europe (this study)	Wild	H1 (1)	E1 (C)	this study
HU17	Hungary	Central Europe (this study)	Wild	H1 (1)	E1 (C)	this study
HU19	Hungary	Central Europe (this study)	Wild	H1 (1)	E1 (C)	this study
HU28	Hungary	Central Europe (this study)	Wild	H1 (1)	E1 (C)	this study
HU54	Hungary	Central Europe (this study)	Wild	H1 (1)	E1 (C)	this study
HU70	Hungary	Central Europe (this study)	Wild	H1 (1)	E1 (C)	this study
HU74	Hungary	Central Europe (this study)	Wild	H1 (1)	E1 (C)	this study
HU81	Hungary	Central Europe (this study)	Wild	H1 (1)	E1 (C)	this study
POL00078	Poland	Central Europe (this study)	Wild	H1 (1)	E1 (C)	this study

POL00222	Poland	Central Europe (this study)	Wild	H1 (1)	E1 (C)	this study
POL00241	Poland	Central Europe (this study)	Wild	H1 (1)	E1 (C)	this study
POL00278	Poland	Central Europe (this study)	Wild	H1 (1)	E1 (C)	this study
POL00285	Poland	Central Europe (this study)	Wild	H1 (1)	E1 (C)	this study
POL00306	Poland	Central Europe (this study)	Wild	H1 (1)	E1 (C)	this study
POL040	Poland	Central Europe (this study)	Wild	H1 (1)	E1 (C)	this study
POL043	Poland	Central Europe (this study)	Wild	H1 (1)	E1 (C)	this study
POL065	Poland	Central Europe (this study)	Wild	H1 (1)	E1 (C)	this study
POL090	Poland	Central Europe (this study)	Wild	H1 (1)	E1 (C)	this study
POL102	Poland	Central Europe (this study)	Wild	H1 (1)	E1 (C)	this study
POL123	Poland	Central Europe (this study)	Wild	H1 (1)	E1 (C)	this study
POL155	Poland	Central Europe (this study)	Wild	H1 (1)	E1 (C)	this study
POL163	Poland	Central Europe (this study)	Wild	H1 (1)	E1 (C)	this study
POL213	Poland	Central Europe (this study)	Wild	H1 (1)	E1 (C)	this study
POL309	Poland	Central Europe (this study)	Wild	H1 (1)	E1 (C)	this study
POL320	Poland	Central Europe (this study)	Wild	H1 (1)	E1 (C)	this study
POL328	Poland	Central Europe (this study)	Wild	H1 (1)	E1 (C)	this study
POL329	Poland	Central Europe (this study)	Wild	H1 (1)	E1 (C)	this study
POL347	Poland	Central Europe (this study)	Wild	H1 (1)	E1 (C)	this study
POL358	Poland	Central Europe (this study)	Wild	H1 (1)	E1 (C)	this study
POL392	Poland	Central Europe (this study)	Wild	H1 (1)	E1 (C)	this study
POL410	Poland	Central Europe (this study)	Wild	H1 (1)	E1 (C)	this study
POL415	Poland	Central Europe (this study)	Wild	H1 (1)	E1 (C)	this study
POL422	Poland	Central Europe (this study)	Wild	H1 (1)	E1 (C)	this study
POL423	Poland	Central Europe (this study)	Wild	H1 (1)	E1 (C)	this study
POL425	Poland	Central Europe (this study)	Wild	H1 (1)	E1 (C)	this study
POL429	Poland	Central Europe (this study)	Wild	H1 (1)	E1 (C)	this study
POL430	Poland	Central Europe (this study)	Wild	H1 (1)	E1 (C)	this study
POL433	Poland	Central Europe (this study)	Wild	H1 (1)	E1 (C)	this study
POL436	Poland	Central Europe (this study)	Wild	H1 (1)	E1 (C)	this study
POL437	Poland	Central Europe (this study)	Wild	H1 (1)	E1 (C)	this study
POL438	Poland	Central Europe (this study)	Wild	H1 (1)	E1 (C)	this study
POL439	Poland	Central Europe (this study)	Wild	H1 (1)	E1 (C)	this study
POL441	Poland	Central Europe (this study)	Wild	H1 (1)	E1 (C)	this study
POL443	Poland	Central Europe (this study)	Wild	H1 (1)	E1 (C)	this study
POL444	Poland	Central Europe (this study)	Wild	H1 (1)	E1 (C)	this study
POL445	Poland	Central Europe (this study)	Wild	H1 (1)	E1 (C)	this study
POL448	Poland	Central Europe (this study)	Wild	H1 (1)	E1 (C)	this study
POL449	Poland	Central Europe (this study)	Wild	H1 (1)	E1 (C)	this study
POL453	Poland	Central Europe (this study)	Wild	H1 (1)	E1 (C)	this study

POL458	Poland	Central Europe (this study)	Wild	H1 (1)	E1 (C)	this study
POL459	Poland	Central Europe (this study)	Wild	H1 (1)	E1 (C)	this study
POL460	Poland	Central Europe (this study)	Wild	H1 (1)	E1 (C)	this study
POL461	Poland	Central Europe (this study)	Wild	H1 (1)	E1 (C)	this study
POL463	Poland	Central Europe (this study)	Wild	H1 (1)	E1 (C)	this study
POL465	Poland	Central Europe (this study)	Wild	H1 (1)	E1 (C)	this study
POL466	Poland	Central Europe (this study)	Wild	H1 (1)	E1 (C)	this study
POL467	Poland	Central Europe (this study)	Wild	H1 (1)	E1 (C)	this study
POL469	Poland	Central Europe (this study)	Wild	H1 (1)	E1 (C)	this study
POL471	Poland	Central Europe (this study)	Wild	H1 (1)	E1 (C)	this study
POL474	Poland	Central Europe (this study)	Wild	H1 (1)	E1 (C)	this study
POL476	Poland	Central Europe (this study)	Wild	H1 (1)	E1 (C)	this study
POL477	Poland	Central Europe (this study)	Wild	H1 (1)	E1 (C)	this study
POL479	Poland	Central Europe (this study)	Wild	H1 (1)	E1 (C)	this study
POL480	Poland	Central Europe (this study)	Wild	H1 (1)	E1 (C)	this study
POL486	Poland	Central Europe (this study)	Wild	H1 (1)	E1 (C)	this study
POL487	Poland	Central Europe (this study)	Wild	H1 (1)	E1 (C)	this study
POL488	Poland	Central Europe (this study)	Wild	H1 (1)	E1 (C)	this study
POL489	Poland	Central Europe (this study)	Wild	H1 (1)	E1 (C)	this study
POL490	Poland	Central Europe (this study)	Wild	H1 (1)	E1 (C)	this study
POL492	Poland	Central Europe (this study)	Wild	H1 (1)	E1 (C)	this study
POL496	Poland	Central Europe (this study)	Wild	H1 (1)	E1 (C)	this study
RUS02	Russia	Eastern Europe (this study)	Wild	H1 (1)	E1 (C)	this study
RUS09	Russia	Eastern Europe (this study)	Wild	H1 (1)	E1 (C)	this study
RUS14	Russia	Eastern Europe (this study)	Wild	H1 (1)	E1 (C)	this study
RUS17	Russia	Eastern Europe (this study)	Wild	H1 (1)	E1 (C)	this study
RUS20	Russia	Eastern Europe (this study)	Wild	H1 (1)	E1 (C)	this study
RUS24	Russia	Eastern Europe (this study)	Wild	H1 (1)	E1 (C)	this study
RUS27	Russia	Eastern Europe (this study)	Wild	H1 (1)	E1 (C)	this study
UA06	Ukraine	Eastern Europe (this study)	Wild	H1 (1)	E1 (C)	this study
UA07	Moldova	Eastern Europe (this study)	Wild	H1 (1)	E1 (C)	this study
UA08	Ukraine	Eastern Europe (this study)	Wild	H1 (1)	E1 (C)	this study
UA09	Ukraine	Eastern Europe (this study)	Wild	H1 (1)	E1 (C)	this study
UA10	Ukraine	Eastern Europe (this study)	Wild	H1 (1)	E1 (C)	this study
UA11	Ukraine	Eastern Europe (this study)	Wild	H1 (1)	E1 (C)	this study
UA13	Ukraine	Eastern Europe (this study)	Wild	H1 (1)	E1 (C)	this study
UA20	Ukraine	Eastern Europe (this study)	Wild	H1 (1)	E1 (C)	this study
POL481	Poland	Central Europe (this study)	Wild	H1 (13)	E1 (C)	this study
BEL158	Belarus	Eastern Europe (this study)	Wild	H1 (8)	E1 (C)	this study
BEL166	Belarus	Eastern Europe (this study)	Wild	H1 (8)	E1 (C)	this study

RUS15	Russia	Eastern Europe (this study)	Wild	H1 (8)	E1 (C)	this study
UA02	Ukraine	Eastern Europe (this study)	Wild	H1 (8)	E1 (C)	this study
UA03	Ukraine	Eastern Europe (this study)	Wild	H1 (8)	E1 (C)	this study
UA12	Ukraine	Eastern Europe (this study)	Wild	H1 (8)	E1 (C)	this study
POL427	Poland	Central Europe (this study)	Wild	H10(11)	E1 (C)	this study
BEL127	Belarus	Eastern Europe (this study)	Wild	H115 (7)	E1 (C)	this study
HU51	Hungary	Central Europe (this study)	Wild	H115 (7)	E1 (C)	this study
RUS01	Russia	Eastern Europe (this study)	Wild	H115 (7)	E1 (C)	this study
UA04	Ukraine	Eastern Europe (this study)	Wild	H115 (7)	E1 (C)	this study
HU02	Hungary	Central Europe (this study)	Wild	H116 (9)	E1 (C)	this study
HU18	Hungary	Central Europe (this study)	Wild	H117 (10)	E1 (C)	this study
POL431	Poland	Central Europe (this study)	Wild	H118 (12)	E1 (C)	this study
BEL112	Belarus	Eastern Europe (this study)	Wild	H67 (5)	E1 (C)	this study
BEL129	Belarus	Eastern Europe (this study)	Wild	H67 (5)	E1 (C)	this study
BEL133	Belarus	Eastern Europe (this study)	Wild	H67 (5)	E1 (C)	this study
BEL162	Belarus	Eastern Europe (this study)	Wild	H67 (5)	E1 (C)	this study
POL00059	Poland	Central Europe (this study)	Wild	H67 (5)	E1 (C)	this study
POL00067	Poland	Central Europe (this study)	Wild	H67 (5)	E1 (C)	this study
POL00220	Poland	Central Europe (this study)	Wild	H67 (5)	E1 (C)	this study
POL00250	Poland	Central Europe (this study)	Wild	H67 (5)	E1 (C)	this study
POL00301	Poland	Central Europe (this study)	Wild	H67 (5)	E1 (C)	this study
POL162	Poland	Central Europe (this study)	Wild	H67 (5)	E1 (C)	this study
POL413	Poland	Central Europe (this study)	Wild	H67 (5)	E1 (C)	this study
POL416	Poland	Central Europe (this study)	Wild	H67 (5)	E1 (C)	this study
POL417	Poland	Central Europe (this study)	Wild	H67 (5)	E1 (C)	this study
POL418	Poland	Central Europe (this study)	Wild	H67 (5)	E1 (C)	this study
POL419	Poland	Central Europe (this study)	Wild	H67 (5)	E1 (C)	this study
POL420	Poland	Central Europe (this study)	Wild	H67 (5)	E1 (C)	this study
POL428	Poland	Central Europe (this study)	Wild	H67 (5)	E1 (C)	this study
POL454	Poland	Central Europe (this study)	Wild	H67 (5)	E1 (C)	this study
POL456	Poland	Central Europe (this study)	Wild	H67 (5)	E1 (C)	this study
POL457	Poland	Central Europe (this study)	Wild	H67 (5)	E1 (C)	this study
POL464	Poland	Central Europe (this study)	Wild	H67 (5)	E1 (C)	this study
POL468	Poland	Central Europe (this study)	Wild	H67 (5)	E1 (C)	this study
POL473	Poland	Central Europe (this study)	Wild	H67 (5)	E1 (C)	this study
POL482	Poland	Central Europe (this study)	Wild	H67 (5)	E1 (C)	this study
POL483	Poland	Central Europe (this study)	Wild	H67 (5)	E1 (C)	this study
POL493	Poland	Central Europe (this study)	Wild	H67 (5)	E1 (C)	this study
BEL119	Belarus	Eastern Europe (this study)	Wild	H68 (6)	E1 (C)	this study
BEL121	Belarus	Eastern Europe (this study)	Wild	H68 (6)	E1 (C)	this study

BEL126	Belarus	Eastern Europe (this study)	Wild	H68 (6)	E1 (C)	this study
BEL134	Belarus	Eastern Europe (this study)	Wild	H68 (6)	E1 (C)	this study
BEL137	Belarus	Eastern Europe (this study)	Wild	H68 (6)	E1 (C)	this study
BEL138	Belarus	Eastern Europe (this study)	Wild	H68 (6)	E1 (C)	this study
BEL140	Belarus	Eastern Europe (this study)	Wild	H68 (6)	E1 (C)	this study
BEL146	Belarus	Eastern Europe (this study)	Wild	H68 (6)	E1 (C)	this study
BEL147	Belarus	Eastern Europe (this study)	Wild	H68 (6)	E1 (C)	this study
BEL150	Belarus	Eastern Europe (this study)	Wild	H68 (6)	E1 (C)	this study
BEL152	Belarus	Eastern Europe (this study)	Wild	H68 (6)	E1 (C)	this study
BEL154	Belarus	Eastern Europe (this study)	Wild	H68 (6)	E1 (C)	this study
HU07	Hungary	Central Europe (this study)	Wild	H68 (6)	E1 (C)	this study
HU16	Hungary	Central Europe (this study)	Wild	H68 (6)	E1 (C)	this study
HU83	Hungary	Central Europe (this study)	Wild	H68 (6)	E1 (C)	this study
POL434	Poland	Central Europe (this study)	Wild	H68 (6)	E1 (C)	this study
POL447	Poland	Central Europe (this study)	Wild	H68 (6)	E1 (C)	this study
POL451	Poland	Central Europe (this study)	Wild	H68 (6)	E1 (C)	this study
POL452	Poland	Central Europe (this study)	Wild	H68 (6)	E1 (C)	this study
POL462	Poland	Central Europe (this study)	Wild	H68 (6)	E1 (C)	this study
POL475	Poland	Central Europe (this study)	Wild	H68 (6)	E1 (C)	this study
POL494	Poland	Central Europe (this study)	Wild	H68 (6)	E1 (C)	this study
RUS03	Russia	Eastern Europe (this study)	Wild	H68 (6)	E1 (C)	this study
RUS05	Russia	Eastern Europe (this study)	Wild	H68 (6)	E1 (C)	this study
RUS16	Russia	Eastern Europe (this study)	Wild	H68 (6)	E1 (C)	this study
RUS25	Russia	Eastern Europe (this study)	Wild	H68 (6)	E1 (C)	this study
RUS26	Russia	Eastern Europe (this study)	Wild	H68 (6)	E1 (C)	this study
BEL111	Belarus	Eastern Europe (this study)	Wild	H75 (4)	E1 (C)	this study
BEL032	Belarus	Eastern Europe (this study)	Wild	H88 (2)	E1 (C)	this study
BEL068	Belarus	Eastern Europe (this study)	Wild	H88 (2)	E1 (C)	this study
BEL072	Belarus	Eastern Europe (this study)	Wild	H88 (2)	E1 (C)	this study
BEL078	Belarus	Eastern Europe (this study)	Wild	H88 (2)	E1 (C)	this study
BEL093	Belarus	Eastern Europe (this study)	Wild	H88 (2)	E1 (C)	this study
BEL102	Belarus	Eastern Europe (this study)	Wild	H88 (2)	E1 (C)	this study
BEL115	Belarus	Eastern Europe (this study)	Wild	H88 (2)	E1 (C)	this study
BEL117	Belarus	Eastern Europe (this study)	Wild	H88 (2)	E1 (C)	this study
BEL128	Belarus	Eastern Europe (this study)	Wild	H88 (2)	E1 (C)	this study
BEL132	Belarus	Eastern Europe (this study)	Wild	H88 (2)	E1 (C)	this study
BEL143	Belarus	Eastern Europe (this study)	Wild	H88 (2)	E1 (C)	this study
BEL149	Belarus	Eastern Europe (this study)	Wild	H88 (2)	E1 (C)	this study
BEL153	Belarus	Eastern Europe (this study)	Wild	H88 (2)	E1 (C)	this study
BEL157	Belarus	Eastern Europe (this study)	Wild	H88 (2)	E1 (C)	this study

BEL161	Belarus	Eastern Europe (this study)	Wild	H88 (2)	E1 (C)	this study
BEL164	Belarus	Eastern Europe (this study)	Wild	H88 (2)	E1 (C)	this study
BEL167	Belarus	Eastern Europe (this study)	Wild	H88 (2)	E1 (C)	this study
POL00069	Poland	Central Europe (this study)	Wild	H88 (2)	E1 (C)	this study
POL00297	Poland	Central Europe (this study)	Wild	H88 (2)	E1 (C)	this study
POL027	Poland	Central Europe (this study)	Wild	H88 (2)	E1 (C)	this study
POL093	Poland	Central Europe (this study)	Wild	H88 (2)	E1 (C)	this study
POL105	Poland	Central Europe (this study)	Wild	H88 (2)	E1 (C)	this study
POL115	Poland	Central Europe (this study)	Wild	H88 (2)	E1 (C)	this study
POL120	Poland	Central Europe (this study)	Wild	H88 (2)	E1 (C)	this study
POL140	Poland	Central Europe (this study)	Wild	H88 (2)	E1 (C)	this study
POL151	Poland	Central Europe (this study)	Wild	H88 (2)	E1 (C)	this study
POL440	Poland	Central Europe (this study)	Wild	H88 (2)	E1 (C)	this study
POL442	Poland	Central Europe (this study)	Wild	H88 (2)	E1 (C)	this study
POL446	Poland	Central Europe (this study)	Wild	H88 (2)	E1 (C)	this study
POL450	Poland	Central Europe (this study)	Wild	H88 (2)	E1 (C)	this study
POL455	Poland	Central Europe (this study)	Wild	H88 (2)	E1 (C)	this study
POL470	Poland	Central Europe (this study)	Wild	H88 (2)	E1 (C)	this study
POL484	Poland	Central Europe (this study)	Wild	H88 (2)	E1 (C)	this study
RUS06	Russia	Eastern Europe (this study)	Wild	H88 (2)	E1 (C)	this study
RUS08	Russia	Eastern Europe (this study)	Wild	H88 (2)	E1 (C)	this study
RUS10	Russia	Eastern Europe (this study)	Wild	H88 (2)	E1 (C)	this study
RUS11	Russia	Eastern Europe (this study)	Wild	H88 (2)	E1 (C)	this study
RUS12	Russia	Eastern Europe (this study)	Wild	H88 (2)	E1 (C)	this study
RUS13	Russia	Eastern Europe (this study)	Wild	H88 (2)	E1 (C)	this study
RUS18	Russia	Eastern Europe (this study)	Wild	H88 (2)	E1 (C)	this study
RUS19	Russia	Eastern Europe (this study)	Wild	H88 (2)	E1 (C)	this study
RUS21	Russia	Eastern Europe (this study)	Wild	H88 (2)	E1 (C)	this study
RUS22	Russia	Eastern Europe (this study)	Wild	H88 (2)	E1 (C)	this study
RUS23	Russia	Eastern Europe (this study)	Wild	H88 (2)	E1 (C)	this study
RUS454	Russia	Eastern Europe (this study)	Wild	H88 (2)	E1 (C)	this study
RUS459	Russia	Eastern Europe (this study)	Wild	H88 (2)	E1 (C)	this study
UA01	Ukraine	Eastern Europe (this study)	Wild	H88 (2)	E1 (C)	this study
UA14	Ukraine	Eastern Europe (this study)	Wild	H88 (2)	E1 (C)	this study
UA17	Ukraine	Eastern Europe (this study)	Wild	H88 (2)	E1 (C)	this study
RUS07	Russia	Eastern Europe (this study)	Wild	H119 (14)	ME	this study
