# Supplementary material for: Contemporary Genetic Structure, Phylogeography and Past Demographic Processes of Wild Boar Sus scrofa Population in Central and Eastern Europe
Source: PLoS One. 2014 Mar 12;9(3):e91401. doi: 10.1371/journal.pone.0091401 (PMC3951376; doi:10.1371/journal.pone.0091401)
Supplement: Table S2 — The haplotypes of wild boar Sus scrofa detected in this study and variable nucleotide positions in relation to a reference sequence from GenBank, accession no. AJ002189. (DOC) [file pone.0091401.s002.doc]

Table S2: The haplotypes of wild boar *Sus scrofa* detected in this study and variable nucleotide positions in relation to a reference sequence from GenBank, accession no. AJ002189.

|  | Nucleotide positions | | | | | | | | | | | | | | | | | | | | | | | | | | | | | | | | | | | | | | | | | | |
| --- | --- | --- | --- | --- | --- | --- | --- | --- | --- | --- | --- | --- | --- | --- | --- | --- | --- | --- | --- | --- | --- | --- | --- | --- | --- | --- | --- | --- | --- | --- | --- | --- | --- | --- | --- | --- | --- | --- | --- | --- | --- | --- | --- |
|  | 15454 | 15469 | 15470 | 15472 | 15477 | 15488 | 15522 | 15543 | 15544 | 15550 | 15555 | 15558 | 15560 | 15562 | 15563 | 15565 | 15566 | 15569 | 15571 | 15578 | 15586 | 15591 | 15614 | 15615 | 15647 | 15706 | 15707 | 15708 | 15710 | 15719 | 15725 | 15737 | 15744 | 15754 | 15818 | 15821 | 15883 | 15932 | 15975 | 16006 | 16049 | 16068 | 16071 |
| Ref. | A | A | A | A | A | T | G | T | G | A | T | A | C | A | T | G | A | C | A | C | C | A | T | T | T | C | A | T | A | A | A | C | A | T | A | C | C | A | A | A | A | G | C |
| H1 | . | . | . | . | . | . | . | . | . | . | . | . | . | . | . | . | . | . | . | . | . | . | . | . | . | A | . | . | . | . | . | . | . | C | . | . | . | . | . | . | . | . | . |
| H2 | . | . | . | . | . | . | . | . | A | . | . | . | . | . | . | . | . | . | . | . | . | . | . | . | C | A | . | . | . | . | . | . | . | C | . | . | . | . | . | . | . | . | . |
| H3 | . | . | . | . | . | . | . | . | . | . | . | T | . | . | . | . | . | . | . | . | . | . | . | . | . | A | . | . | . | . | . | . | . | C | . | . | . | . | . | . | . | . | . |
| H4 | . | . | . | . | . | . | . | . | . | . | . | . | . | . | . | . | . | . | . | . | . | . | . | . | . | A | . | . | . | G | . | . | . | C | . | . | . | . | . | . | . | . | T |
| H5 | . | . | . | . | . | . | . | . | . | . | . | . | . | . | . | . | . | . | . | . | . | . | . | . | . | A | . | . | . | . | . | . | . | C | . | T | . | . | . | . | . | . | . |
| H6 | . | . | . | . | . | . | . | . | . | . | . | . | . | . | . | . | . | . | . | . | . | . | . | . | . | A | . | . | . | . | . | . | . | . | . | . | . | . | . | . | . | . | . |
| H7 | . | . | . | . | . | . | . | . | . | . | . | . | . | . | . | . | . | . | . | . | . | . | . | . | C | A | . | . | . | . | . | . | . | C | . | . | . | . | . | . | . | . | . |
| H8 | . | . | . | . | . | . | . | . | . | . | . | . | . | . | . | . | . | . | . | . | . | . | . | . | . | A | . | . | . | . | . | . | . | C | . | . | . | . | . | . | . | . | T |
| H9 | . | G | C | T | G | . | . | . | . | . | . | . | . | . | . | . | . | . | . | . | . | . | . | . | . | A | . | . | . | . | . | . | . | C | . | . | . | . | . | . | . | . | . |
| H10 | . | . | . | . | . | . | . | . | . | . | . | . | . | . | . | . | . | . | . | . | . | . | . | . | . | A | . | . | . | . | . | . | . | C | . | . | . | . | . | . | T | . | . |
| H11 | . | . | . | . | . | . | . | . | . | . | . | . | . | . | . | . | . | . | . | . | . | . | . | . | . | A | . | . | . | . | . | . | . | . | . | . | . | . | . | . | . | . | . |
| H12 | . | . | . | . | . | . | . | . | . | . | . | . | . | . | . | . | . | . | . | . | . | . | . | . | . | A | . | . | . | . | . | . | . | C | . | . | . | . | . | . | . | . | . |
| H13 | . | . | . | . | . | . | . | . | . | . | . | . | . | . | . | . | . | . | . | . | . | . | . | . | . | A | . | . | . | . | . | . | . | C | . | . | . | . | T | . | . | . | . |
| H14 | . | . | . | . | . | C | A | C | . | C | A | T | A | C | A | T | G | A | C | . | T | . | C | C | C | A | T | A | C | . | G | . | G | . | G | . | . | . | . | G | . | A | . |
| H15 | C | . | . | . | . | . | . | . | . | . | . | . | . | . | . | . | . | . | . | . | . | . | . | . | . | A | . | . | . | . | . | . | . | . | . | . | . | . | . | . | . | . | . |
| H16 | . | . | . | . | . | . | . | C | . | . | . | . | . | . | . | A | . | A | . | T | T | G | C | . | . | A | . | . | . | . | G | T | . | . | . | T | T | G | . | G | . | . | . |
