# Supplementary material for: Contemporary Genetic Structure, Phylogeography and Past Demographic Processes of Wild Boar Sus scrofa Population in Central and Eastern Europe
Source: PLoS One. 2014 Mar 12;9(3):e91401. doi: 10.1371/journal.pone.0091401 (PMC3951376; doi:10.1371/journal.pone.0091401)
Supplement: Table S3 — Results of SAMOVA, ΦCT: fixation index among groups; ΦSC: fixation index among populations within groups; ΦST: fixation index within populations. (DOC) [file pone.0091401.s003.doc]

Table S3. Results of SAMOVA, Φ_CT_: fixation index among groups; Φ_SC_: fixation index among populations within groups; Φ_ST_:fixation index within populations.

| Number of groups (K) | Subdivision | Φ_CT_ | Φ_SC_ | Φ_ST_ |
| --- | --- | --- | --- | --- |
| 2 | (6) (1, 2, 3, 4, 5, 7, 8) | 0.075 | 0.020** | 0.093* |
| **3** | **(6) (8) (1, 2, 3, 4, 5, 7)** | **0.059*** | **0.019*** | **0.077**** |
| 4 | (6) (5) (1, 2, 8) (3, 4, 7) | 0.055* | -0.002 | 0.054** |
| 5 | (6) (5) (1) (2, 8) (3, 4, 7) | 0.058* | -0.009 | 0.049** |
| 6 | (6) (5) (1) (2) (8) (3, 4, 7) | 0.065* | -0.017 | 0.049** |
| 7 | (6) (5) (1) (2) (4) (8) (3, 7) | 0.088* | -0.046 | 0.046** |

* *P* < 0.05; ** *P* < 0.001
